# Identification of a new gene regulatory circuit involving B cell receptor activated signaling using a combined analysis of experimental, clinical and global gene expression data

**DOI:** 10.18632/oncotarget.9219

**Published:** 2016-05-07

**Authors:** Alexandra Schrader, Katharina Meyer, Neele Walther, Ailine Stolz, Maren Feist, Elisabeth Hand, Frederike von Bonin, Maurits Evers, Christian Kohler, Katayoon Shirneshan, Martina Vockerodt, Wolfram Klapper, Monika Szczepanowski, Paul G. Murray, Holger Bastians, Lorenz Trümper, Rainer Spang, Dieter Kube

**Affiliations:** ^1^ Department of Haematology and Medical Oncology, University Medical Centre of the Georg-August University Göttingen, Göttingen, Germany; ^2^ GRK1034 of the Deutsche Forschungsgemeinschaft, Georg-August University Göttingen, Göttingen, Germany; ^3^ Department of Statistical Bioinformatics, Institute for Functional Genomics, University of Regensburg, Regensburg, Germany; ^4^ Goettingen Center for Molecular Biosciences (GZMB) and University Medical Center, Institute of Molecular Oncology, Section for Cellular Oncology, Göttingen, Germany; ^5^ Network Molecular Mechanism of Malignant Lymphoma (MMML) of the Deutsche Krebshilfe, Germany; ^6^ BMBF-Network HämatoSys, Germany; ^7^ BMBF-Network Myc-Sys, Germany; ^8^ School of Cancer Sciences, University of Birmingham, Birmingham, UK; ^9^ University-Hospital Schleswig-Holstein, Hematopathology Section and Lymph Node Registry Kiel, Kiel, Germany; ^10^ Department of Anatomy, University Medical Centre of the Georg-August University Göttingen, Göttingen, Germany; ^11^ Present address: Laboratory of Lymphocyte Signaling and Oncoproteome, Department I of Internal Medicine, University Hospital Cologne, Center for Integrated Oncology (CIO) Köln-Bonn, Cologne, Germany; ^12^ Present address: Department of Anatomy, University Medical Centre of the Georg-August University Göttingen, Göttingen, Germany; ^13^ Current address: The John Curtin School of Medical Research the Australian National University Canberra, Australia

**Keywords:** lymphoma, B cell receptor signaling, guided clustering, cell cycle delay, chromosomal aberrations

## Abstract

To discover new regulatory pathways in B lymphoma cells, we performed a combined analysis of experimental, clinical and global gene expression data. We identified a specific cluster of genes that was coherently expressed in primary lymphoma samples and suppressed by activation of the B cell receptor (BCR) through αIgM treatment of lymphoma cells *in vitro*. This gene cluster, which we called BCR.1, includes numerous cell cycle regulators. A reduced expression of BCR.1 genes after BCR activation was observed in different cell lines and also in CD10^+^ germinal center B cells. We found that BCR activation led to a delayed entry to and progression of mitosis and defects in metaphase. Cytogenetic changes were detected upon long-term αIgM treatment. Furthermore, an inverse correlation of BCR.1 genes with c-Myc co-regulated genes in distinct groups of lymphoma patients was observed. Finally, we showed that the BCR.1 index discriminates activated B cell-like and germinal centre B cell-like diffuse large B cell lymphoma supporting the functional relevance of this new regulatory circuit and the power of guided clustering for biomarker discovery.

## INTRODUCTION

Diffuse large B-cell lymphoma (DLBCL), the most common lymphoid malignancy in adults, is derived from germinal centre or post-germinal centre B cells [1]. DLBCLs are clinically and molecular heterogenous [1–4]. Long-term disease-free survival is now a reality for at least 50 percent of patients. However, approximately 30 percent of patients with DLBCL, mainly those with advanced disease and those who relapse, will not respond appropriately to current treatments [4, 5]. A better understanding of the specific pathways underlying the malignant phenotype in DLBCL is urgently required. This would allow not only the development of novel targeted therapeutic approaches for defined subgroups of DLBCL, but also the identification of new biomarkers [6].

Activation of B lymphocytes is associated with rapid proliferation, somatic hypermutation and targeted DNA double strand breaks in the immunoglobulin (Ig) locus. This process is under tight spatial and temporal control achieved by extracellular signals sensed via B cell receptor (BCR), CD40, Toll-like receptors (TLR), B-cell activating factor (BAFF) receptor, interleukin-21 (IL21) receptor and cell intrinsic mechanisms [1, 7–13]. Molecular signatures suggest that specific signaling networks and survival mechanisms exist in DLBCL, caused either by extracellular signals from the lymphoma microenvironment or related pathway mutations within the lymphoma cells, or both [1, 13].

Based on the cell of origin two main molecularly defined subgroups of DLBCLs can be described: activated B cell (ABC) - like and germinal centre B cell (GCB) – like DLBCL [14]. This cell-of-origin-approach is driven by class labels. In parallel, signatures such as stroma, host response, OxPhos and BCR/proliferation or Jak/STAT and IKK have been described by comprehensive consensus clustering, and identify the lymphoma microenvironment as a defining feature [15–19]. The level of c-Myc activity also allows the subdivision of DLBCL [20–23]; a high c-Myc index or a high number of Myc positive lymphoma cells is associated with shorter survival [20–23], but the subentities defined by Myc expression only partly overlap with the ABC/GCB-like signatures. Although these studies predict activity of different oncogenic pathways for DLBCL subtypes the functional consequences of differential gene expression are still poorly understood [15, 18, 19, 24–27],

Recently we introduced guided clustering as a strategy that integrates experimental, clinical and global gene expression data to investigate aggressive non-Hodgkin lymphoma (NHL) [28–30]. This approach does not utilize the class labels from the clinical data (e.g. disease types or clinical outcomes) to derive a molecular signature, but instead is based on the underlying biology for example the activity of an entire pathway or pathway networks established by cell perturbation experiments. For example, in our recent proof of principle analysis we were able to captured quantitatively a link between BCL-6 gene regulation and TLR signaling. The combined analysis of experimental and global gene expression data can allow the development of functional hypotheses [29, 31]. Therefore, transcriptional modules (groups of coherent expressed genes) can be described as potential major oncogenic hubs that are conserved between patients.

The present study aimed to extract functional information on oncogenic pathway activities in distinct DLBCL cases based on gene expression from primary tumours and multiple *in vitro* interventions. To do this the guided clustering-driven identification of transcriptional modules was extended and multiple single *in vitro* interventions were combined *in silico* to identify transcriptional modules conserved between patients but dominantly affected by for example BCR signaling but not TLR, IL21, CD40L or BAFF. This is to enable the assessment of pathway specific activities in primary lymphoma cases.

A new regulatory circuit in B cells was identified. A group of dominantly suppressed genes upon BCR activation is involved in an overall diminished capacity of the cells to enter mitosis, leading to defects in metaphase as well as increased chromosomal aberrations. In a subgroup of GCB-like DLBCL with low Myc activity this new regulatory circuit is dominant. This regulatory circuit is nearly absent in BL but active in c-Myc^high^ DLBCLs. Our data supports the view that BCR signaling is context dependent and capable not only of promoting cell survival and proliferation but also delaying cell cycle progression thereby potentially increasing chromosomal aberrations. It further underpins the notion that defined pathways stimulated by microenvironmental factors activating the BCR are involved in DLBCL development and that these pathways might be of therapeutic relevance. Our analysis shows how guided clustering lead to the discovery of biomarkers for cancer stratification.

## RESULTS

### A combined analysis of experimental and tumour derived global gene expression data identifies a set of genes specifically suppressed by BCR activation

Ligands activating pattern recognition receptors, BCR, CD40, BAFF-receptors and IL21 receptor are well known mediators of signalling in B cells and important components of the GC B cell reaction. Furthermore, it is well known that elements of the corresponding signalling pathways are mutated in DLBCL [1, 7–13]. Thus, the signalling pathways activated by these factors represent promising candidates for the identification of oncogenic pathway signatures in DLBCL via guided clustering. To answer these questions, as a model cell line, BL2 was chosen. The criteria for their selection were: absence or low pathway activity, a strong signal induction by stimuli, and measurable global gene expression changes suitable for bioinformatic analysis as we have previously described [32].

**Figure 1:**
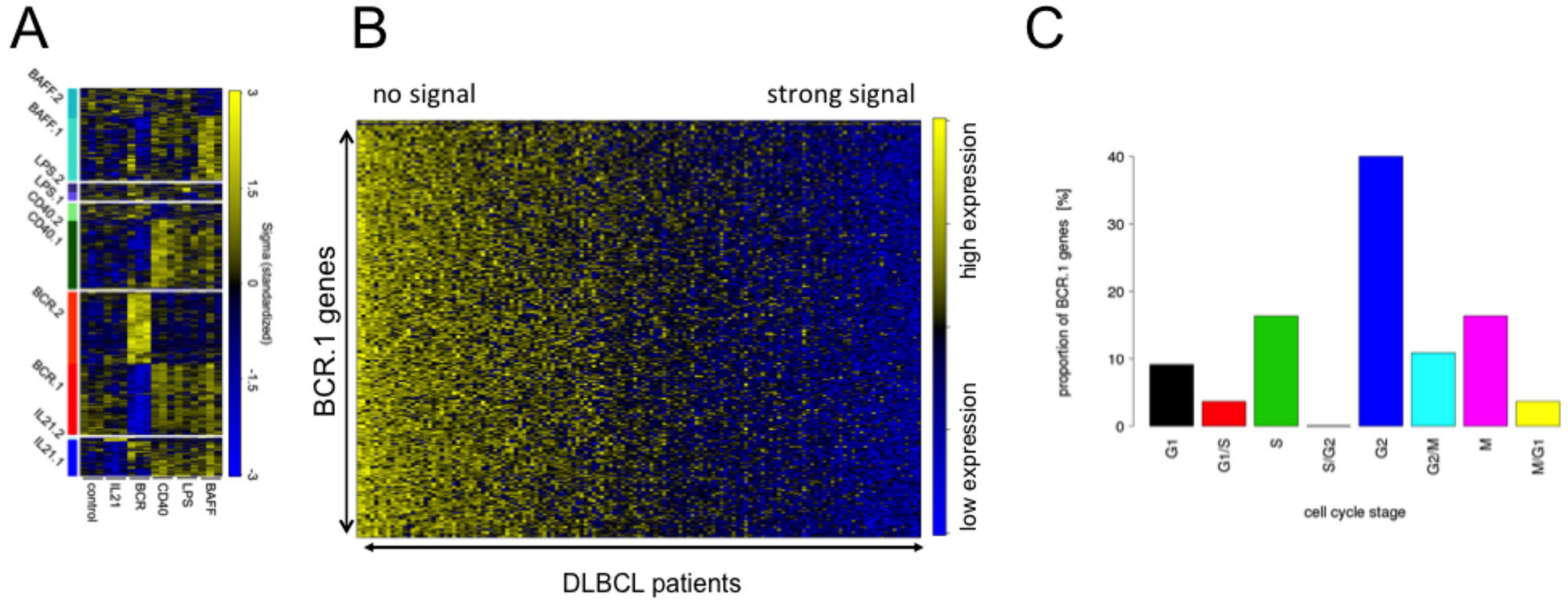
Guided Clustering identifies gene clusters dominantly affected by one specific intervention **A.** Heatmap representation of the gene expression levels for the genes within the ten transcriptional modules identified by guided clustering analysis. Global gene expression of stimulated BL2 cells and gene expression profiles from 175 lymphoma patients without Myc-translocations [28, 30]. BL2 cells treated with αIgM treatment, CD40L, LPS, BAFF and IL21. Each column in the heatmap represents a gene and each row represents a microarray sample. Yellow and blue indicate high and low gene expression. Heatmap shows the gene expression of the corresponding cluster genes in stimulated BL2 cells compared to unstimulated cells. **B.** A heatmap representation of BCR.1 genes in gene expression profiles of 137 primary lymphoma. The patient samples are ordered according to their increasing BCR.1 index starting with the lowest index on the very left end of the heatmap [30]. **C.** Gene ontology based analysis of the fraction of genes from the BCR.1 gene cluster associated with the cell cycle. GO Term analysis gives frequency of BCR.1 genes involved in different cell cycle phases (information taken from www.cyclebase.org)(for additional details see also Supplementary Table S2).

Microarray data sets obtained from human transformed germinal centre B cells (BL2) stimulated with CD40L, BAFF, IL21, αIgM F(ab)_2_ fragments and lipopolysaccharide (LPS) were processed as described previously, combined, and analysed by guided clustering using large-scale gene expression data from 175 DLBCL patients [28, 32]. The patients were selected from the MMML-cohort and are representative of non-mBLs without chromosomal *MYC* translocations [30]. Guided clustering was performed in the following way: the guiding datasets were obtained from *in vitro* stimulated BL2 cells and only genes driven dominantly by one stimuli, but not the others, included. These data sets were integrated with gene expression profiles of primary lymphoma material. Ten different gene clusters were identified characterized by increased or suppressed gene expression in experiments and concordantly expressed in lymphoma patients: CD40.1, CD40.2, IL21.1, IL21.2, BAFF.1, BAFF.2, BCR.1, BCR.2, LPS.1 and LPS.2 (Figure 1A, Table I). The suffix “.1” denotes genes mainly suppressed and “.2” those genes mainly activated (Table I, Supplementary Table S1). These clusters most likely represent surrogates of pathway activity dominated by one of the stimuli. To delineate so far undescribed biological outcomes the following experiments were focused on αIgM driven suppression of gene expression.

**Table I:** Identification of different clusters of genes displaying a coherent expression across patient profiles affected by multiple *in vitro* interventions using guided clustering

intervention	Index name	Number of genes coherently expressed in DLBCL	Gene expression changes in BL2 cells
αIgM			
	BCR.1	288	suppressed
	BCR.2	286	activated
CD40L			
	CD40.1	288	suppressed
	CD40.2	71	activated
BAFF			
	BAFF.1	255	suppressed
	BAFF.2	122	activated
IL21			
	IL21.1	148	suppressed
	IL21.2	13	activated
LPS			
	LPS.1	34	suppressed
	LPS.2	37	activated

Next the 137 DLBCL patient samples used for guided clustering were sorted according to an individual index value derived from BCR.1. This index reflects the extent to which BCR.1 genes are expressed within an individual sample. A high BCR.1 index indicates strong down-regulation of the BCR.1 genes. Finally, lymphoma samples were sorted according to their individual index (Figure 1B). This results in a spectrum of lymphoma samples ranging from low to high BCR.1 index scores. A substantial number of analysed patient samples is characterized by a corresponding strong BCR.1 signal.

Molecular functions, biological processes, cellular components and pathways of BCR.1 genes were identified by gene ontology (GO) based gene set enrichment analyses (Table II, Supplementary Table S2). Among the top 100 BCR.1 genes, the most significantly enriched gene sets corresponded to biological processes which included cell cycle checkpoints, microtubule-based processes, microtubule cytoskeleton organization, spindle organization, mitotic chromosome segregation or sister chromatid segregation as well as response to DNA damage stimuli, organelle organization or metabolic processes (Table II). In Figure 1C BCR.1 index genes associated with cell cycle regulatory functions are presented. Genes are grouped according to the corresponding cell cycle phases in which they are involved, demonstrating that more than 40% of cell cycle regulatory genes within this gene cluster belong to G2 and M cell cycle regulation.

**Table II:** Genes of coherent expression across patient profiles reflecting the activity of a branch of B cell receptor signaling

1	XPO1	50	RFC3	99	CLINT1	148	SFRS4	197	BBS10	246	MRPL49
2	PMS1	51	ECT2	100	DLAT	149	HSPH1	198	ZNF638	247	CSE1L
3	RNASEN	52	DSCC1	101	RIOK2	150	FOSL1	199	SRBD1	248	PLA2G12A
4	LBR	53	C12orf48	102	CKAP2	151	KIAA0406	200	GTF2E1	249	ELP4
5	CETN3	54	WRAP53	103	NARS2	152	DSN1	201	MEN1	250	UBTF
6	DCK	55	PARP2	104	CEP76	153	PRPF4B	202	LSM2	251	GTF3C1
7	GPN3	56	TRIP13	105	CACYBP	154	C13orf34	203	SRPK1	252	BCOR
8	SLBP	57	KIF11	106	TMEM97	155	BAG2	204	TTRAP	253	C5orf22
9	RHOT1	58	AASDHPPT	107	POLE2	156	ZNHIT3	205	ZNF184	254	TTC33
10	CAND1	59	FARSA	108	USP13	157	PDHB	206	MICB	255	PSME4
11	RACGAP1	60	CTCF	109	RAD1	158	CCDC56	207	TUBD1	256	PPM1B
12	KIF20A	61	RNASEH2A	110	GCDH	159	CUTC	208	NFYB	257	PIAS4
13	AURKA	62	NEIL3	111	CPOX	160	C14orf104	209	KIF18B	258	WBP4
14	HMMR	63	STIL	112	CLPX	161	COX11	210	PRDM10	259	ACAP2
15	NDC80	64	TARDBP	113	MORC2	162	CDK8	211	HMGN4	260	GRSF1
16	CDC20	65	ARMC1	114	RFC5	163	C8orf41	212	RAD54B	261	GADD45GIP1
17	CENPA	66	MRPL35	115	RAD51C	164	AURKAIP1	213	DTWD1	262	MSH2
18	PLK1	67	WDR67	116	RCN2	165	IMP3	214	BRD8	263	DTYMK
19	NEK2	68	NUP37	117	PIGF	166	MINA	215	TRMT61B	264	FRAT2
20	CCNA2	69	MRPL12	118	ACTR6	167	PUM2	216	RNF34	265	STAMBP
21	KIF18A	70	RAD54L	119	CDC73	168	DHX29	217	CDC27	266	C17orf75
22	RMI1	71	C16orf53	120	MDM1	169	COASY	218	EXOC1	267	FANCL
23	PBK	72	ALG6	121	KIAA0528	170	THAP7	219	SACM1L	268	SFRS2B
24	PRC1	73	TROAP	122	PARG	171	MRPS34	220	VPS33B	269	HMBS
25	BUB1B	74	CDC7	123	THAP11	172	CCDC51	221	SUCLA2	270	PAAF1
26	PLK4	75	RFC4	124	C12orf52	173	MRPL17	222	MRS2	271	NAA40
27	CDCA8	76	UNG	125	PTCD3	174	COIL	223	C6orf211	272	NDUFS3
28	NCAPH	77	PPAT	126	CASP6	175	ATMIN	224	HMGB3	273	DUT
29	TMEM48	78	FASTKD1	127	CTR9	176	SMARCAL1	225	GEMIN6	274	STRA13
30	OIP5	79	KIF15	128	MRPL46	177	COQ9	226	MRPS16	275	HADH
31	CEP55	80	ANP32A	129	GPSM2	178	COBRA1	227	MCM10	276	SEPHS1
32	KIF14	81	SRRD	130	NDUFC1	179	MED20	228	AP1AR	277	ABHD10
33	ESPL1	82	LRRC47	131	UBE2G1	180	CCDC99	229	C4orf27	278	SLC4A1AP
34	POLA2	83	PREB	132	PRPSAP1	181	SIP1	230	FASTKD3	279	STRADA
35	FEN1	84	ZC3H14	133	NCAPD3	182	FANCG	231	CLCN3	280	CBX1
36	BRCA1	85	TTK	134	DPF2	183	MCM2	232	PRMT5	281	MRPS27
37	TUBG1	86	EFTUD1	135	HEATR3	184	CRIPT	233	TDP1	282	WRB
38	MRPL16	87	OSBPL11	136	SHCBP1	185	GAPVD1	234	LARS2	283	DERA
39	TACC3	88	MYCBP	137	C4orf41	186	CNP	235	BARD1	284	SAR1B
40	SAC3D1	89	DYNLL1	138	SKP2	187	RAB11A	236	MSH3	285	MAP3K4
41	ASPM	90	NIF3L1	139	MTX2	188	MRPS31	237	MPHOSPH6	286	KIAA1279
42	WDHD1	91	MRFAP1L1	140	MUDENG	189	MRPL34	238	MTIF2	287	PPCS
43	BIRC5	92	NARG2	141	C15orf44	190	PHB	239	ATR	288	C5orf15
44	CCNB1	93	SPAST	142	GINS1	191	POP4	240	PPP2R5E		
45	ASF1B	94	ZW10	143	ZWILCH	192	AGGF1	241	PSMC6		
46	ORC1L	95	ELF2	144	MRPL18	193	KIF22	242	ZNF107		
47	KIF2C	96	ADH5	145	PEX14	194	POP7	243	EIF2B4		
48	CDCA3	97	ORC4L	146	TOMM70A	195	DDX23	244	C9orf40		
49	FOXM1	98	ORC2L	147	TSN		ZBED5	245	RTF1		

Shown are the genes of the newly identified transcriptional module called BCR.1. For all transcriptional modules please refer to TABLE E1.

### Verification and validation of gene expression after αIgM treatment reveals the downregulation of BCR.1 index genes

Table III summarises the fold changes in expression of genes from the BCR.1 cluster involved in cell cycle regulation that were suppressed by αIgM treatment of BL2 cells.

**Table III:** Genes involved in different aspects of cell cycle regulation as described by guided clustering for BCR.1

	Gene symbol	Name	Function in cell cycle and related processes	Fold change (log_2_FC)	Fold change (log_2_FC)
				BL2	CD10+ tonsilar B cells
1	ASPM	asp (abnormal spindle) homolog	role in mitotic spindle regulation and coordination of mitotic processes	−0,51	−0,45
2	ATMIN	ATM interactor	ATM/ATR-substrate CHEK2-interacting zinc finger protein; Plays a crucial role in cell survival and RAD51 foci formation in response to methylating DNA damage. Involved in regulating the activity of ATM in the absence of DNA damage	−0,39	n.a.
3	AURKA	Aurora-kinase A	Mitotic serine/threonine kinases that contributes to the regulation of mitosis	−0,68	−1,03
4	BARD1	BRCA1 associated RING domain 1	Constitutes together with BRCA1 an Ubiquitin E3 ligase	−0,68	−0,53
5	BCOR	BCL6 corepressor	Transcriptional corepressor	−0,69	−0,43
6	BIRC5/Survivin	baculoviral IAP repeat containing 5	Component of the chromosomal passenger complex (CPC), a complex that acts as a key regulator of mitosis	−0,45	−0,44
7	BRCA1	breast and ovarian cancer susceptibility protein 1	Constitutes together with BARD1 an ubiquitin E3 ligase. Involved in DNA repair and in the regulation of mitosis.	−0,44	−0,32
8	BUB1B/MAD3L/BUBR1	budding uninhibited by benzimidazoles 1 homolog beta	Essential component of the mitotic spindle assembly checkpoint	−0,37	−0,608
9	C12orf52	RBP-J interacting and tubulin associated	Tubulin-binding protein that acts as a negative regulator of Notch signaling pathway	−0,83	n.a.
10	C13orf34/bora	aurora kinase A activator	Required for the activation of AURKA at the onset of mitosis	−0,42	−0,417
11	CCNA2	Cyclin A2	binds and activates CDK1 or CDK2 kinases, and thus promotes both cell cycle G1/S and G2/M transitions	−0,42	−0,57
12	CCNB1	Cyclin B1	Binds and activates CDK1, Eessential for the control of the cell cycle at the G2/M (mitosis) transition	−0,67	−0,67
13	CDC20	cell division cycle 20 homolog	In metaphase the MAD2L1-CDC20-APC/C ternary complex is inactive and in anaphase the CDC20-APC/C binary complex is active in degrading substrates	−0,57	−0,87
14	CDC27	Anaphase-promoting complex subunit 3	Subunit of the APC/C, cell cycle-regulated E3 ubiquitin ligase that controls progression through mitosis and the G1 phase	−0,27	−0,52
15	CDC7	cell division cycle 7 homolog	G1/S phase transition	−0,35	−0,70
16	CDCA3	Trigger of mitotic entry protein 1	F-box-like protein which is required for entry into mitosis. Acts by participating in E3 ligase complexes	−0,54	−0,66
17	CENPA	centromere protein A	Histone-like protein, Required for recruitment and assembly of kinetochore proteins, mitotic progression and chromosome segregation	−0,52	−1,05
18	CETN3	centrin	located at the centrosome of interphase and mitotic cells, where it plays a fundamental role in centrosome duplication	−0,99	−0,67
19	COBRA1	cofactor of BRCA1	Essential component of the NELF complex, a complex that negatively regulates the elongation of transcriptionby RNA polymerase II	−0,37	n.a.
	COIL	coilin	During mitosis, CBS disassemble, coinciding with a mitotic-specific phosphorylation of p80coilin	n.a	−0,23
20	CSE1L	CSE1 chromosome segregation 1-like	may play a role both in apoptosis and in cell proliferation	−0,34	−0,37
21	DSCC1	Defective in sister chromatid cohesion protein 1	couple DNA replication to sister chromatid cohesion through regulation of the acetylation of the cohesin subunit SMC3	−0,55	−0,37
22	DSN1	MIND kinetochore complex component, homolog (S. cerevisiae)	MIND kinetochore complex component	−0,69	−0,41
23	ECT2/ARH-GEF31	Epithelial cell-transforming sequence 2 oncogene	Required for signal transduction pathways involved in the regulation of cytokinesis	−0,31	−0,70
24	ESPL1	extra spindle pole bodies homolog 1	Caspase-like protease, which plays a central role in the chromosome segregation by cleaving the SCC1/RAD21 subunit of the cohesin complex at the onset of anaphase	−0,35	−0,42
25	FANCG	Fanconi anemia, complementation group L	maintenance of normal chromosome stability	−0,51	−0,42
26	FANCL	Fanconi anemia, complementation group L	mediates monoubiquitination of FANCD2, a key step in the DNA damage pathway	−0,72	−0,65
27	FOSL1/FRA1	FOS-like antigen 1	regulators of cell proliferation	−0,28	+0,22
28	FOXM1	forkhead box M1	Transcriptional factor regulating the expression of cell cycle genes essential for DNA replication and mitosis	n.a	−0,33
29	FRAT2	frequently rearranged in advanced T-cell lymphomas 2	Positively regulates the Wnt signaling pathway by stabilizing beta-catenin through the association with GSK-3	−0,96	n.a.
30	GADD45GIP1		growth arrest and DNA-damage-inducible, gamma interacting protein 1	−1,56	n.a.
31	HMMR/RHAMM	hyaluronan-mediated motility receptor	Receptor protein and associated with mitotic spindles	−0,86	−0,99
32	KIAA0406	TELO2 interacting protein 1	Regulator of the DNA damage response	−0,32	n.a.
33	KIF11/KSP/Eg5	kinesin family member 11	Motor protein required for establishing a bipolar spindle.	n.a	−0,71
34	KIF14	kinesin family member 14	Plays an essential role in cytokinesis	−0,45	−0,75
	KIF15	HKLP2, kinesin family member 15	Plus-end directed kinesin-like motor enzyme involved in mitotic spindle assembly	−0,67	−0,51
35	KIF18A	kinesin family member 18A	Microtubule-depolymerizing kinesin which plays a role in chromosome congression.	−0,56	−0,38
36	KIF18B	Kinesin family member 18B	Microtubule-depolymerizing kinesin	n.a	−0,49
37	KIF20A	Rab6-interacting kinesin-like protein	Mitotic kinesin required for chromosome passenger complex (CPC)-mediated cytokinesis	−1,65	−1,26
38	KIF22	Kinesin family member 22	movements of chromosomes during mitosis	−0,36	−0,27
39	KIF2C/MCAK	Mitotic centromere-associated kinesin	Microtubule depolymerase, Regulates the turnover of microtubules at kinetochores	n.a	−0,36
40	MINA	MYC induced nuclear antigen	Involved in cellular proliferation	−0,64	n.a.
41	MYCBP	MYC-binding protein	May control the transcriptional activity of MYC	−0,33	−0,97
42	NEK2	NIMA (never in mitosis gene a)-related kinase 2	control of centrosome separation and bipolar spindle formation in mitotic cells	−0,64	−0,58
43	NCAPD3	non-SMC condensin II complex, subunit D3	Regulatory subunit of the condensin-2 complex, a complex which establishes mitotic chromosome architecture and is involved in physical rigidity of the chromatid axis	n.a	−0,24
44	NCAPH	non-SMC condensin I complex, subunit H	Regulatory subunit of the condensin complex, a complex required for conversion of interphase chromatin into mitotic-like condense chromosomes	n.a	−0,43
45	NDC80	NDC80 homolog	kinetochore complex component	n.a	−0,97
46	OIP5	Opa interacting protein 5	Required for recruitment of CENPA to centromeres and normal chromosome segregation during mitosis	−0,61	−0,74
47	PARP2	poly (ADP-ribose) polymerase 2	Involved in the base excision repair (BER) pathway,	−0,33	−0,49
48	PBK/TOPK	PDZ-binding kinase	Phosphorylates MAP kinase p38, active only in mitosis. May also play a role in the activation of lymphoid cells. When phosphorylated, forms a complex with TP53, leading to TP53 destabilization and attenuation of G2/M checkpoint during doxorubicin-induced DNA damage	−0,66	−0,51
49	PLK1	Polo-like kinase 1	critical regulatorsof cell cycle progression, mitosis, cytokinesis, and the DNA damage response	−0,78	−0,29
50	PLK4/STK18	Polo-like kinase 4	able to induce centrosome amplification through the simultaneous generation of multiple procentrioles adjoining each parental centriole during S phase. Phosphorylates CDC25C and CHEK2	−0,27	−0,31
51	PMS1	DNA mismatch repair protein	involved in the repair of mismatches in DNA	−1,24	n.a.
52	PRC1	protein regulator of cytokinesis 1	Required for KIF14 localization to the central spindle and midbody.	−0,57	−0,63
53	PRDM10	PR-domain family member 7	transcriptional regulation, involved in B cell differentiation and tumour suppression	−1,32	n.a.
54	PUM2	pumilio homolog 2	Sequence-specific RNA-binding protein, support proliferation andself-renewal of stem cells	−0,32	n.a.
55	RAD1	Rad1-like DNA damage checkpoint protein	cell cycle checkpoint protein	−0,28	n.a.
56	FANCO/RAD51C	RAD51 homolog C	early function in DNA repair in facilitating phosphorylation of the checkpoint kinase CHEK2 and thereby transduction of the damage signal, leading to cell cycle arrest and HR activation	−0,32	n.a.
57	RAD54B	DNA repair and recombination protein RAD54B	Involved in DNA repair and mitotic recombination	−0,96	−0,45
58	RAD54L	DNA repair and recombination protein RAD54-like	Involved in DNA repair and mitotic recombination	−0,49	−0,23
59	RFC3	replication factor C (activator 1) 3	elongation of primed DNA templates by DNA polymerase delta and epsilon requires the action of the accessory proteins proliferating cell nuclear antigen (PCNA) and activator 1	−0,53	n.a.
60	RMI1	RecQ mediated genome instability 1	important role in the processing ofhomologous recombination intermediates to limit DNA crossover formation in cells	−0,54	n.a.
61	RNASEN	drosha, ribonuclease type III	is involved in the initial step ofmicroRNA (miRNA) biogenesis	−0,71	n.a.
62	SAC3D1	SAC3 domain containing 1	Involved in centrosome duplication and mitotic progression	−1,05	−1,01
63	SKP2	S-phase kinase-associated protein 2 - E3 ubiquitin protein ligase	subunit of the SCF ubiquitin ligase	−0,95	
64	SPAST	spastin	completion of the abscission stage of cytokinesis	−0,83	−0,53
65	STIL	SCL/TAL1 interrupting locus	its long-term silencing affects cell survival and cell cycle distribution as well as decreases CDK1 activity correlated with reduced phosphorylation of CDK1	n.a	−0,43
66	STRADA	STE20-related kinase adaptor alpha	necessary for STK11-induced G1 cell cycle arrest	−0,48	−0,35
67	STRA13/FANCM/CENP-X	Fanconi anemia-associated polypeptide	involved in DNA damage repair and genome maintenance	−0,38	−0,275
68	TACC3	transforming, acidic coiled-coil containing protein 3	microtubule-associated adaptor protein	−0,41	−0,51
69	TTK/MPS1	Phosphotyrosine picked threonine-protein kinase	Essential for chromosome alignment by enhancing AURKB activity (via direct CDCA8 phosphorylation) at the centromere, and for the mitotic checkpoint	−0,37	−0,45
70	TUBG1	Tubulin G1	major constituent of microtubules,	−0,61	n.a.
71	UBE2G1	ubiquitin-conjugating enzyme E2G 1	member of theE2 ubiquitin-conjugating enzyme family and catalyzes the covalent attachment of ubiquitin to other proteins	−0,77	−0,55
72	ZW10	kinetochore associated, homolog	Essential component of the mitotic spindle assembly checkpoint	−0,305	n.a.

Data are extracted from U133 plus 2.0 microarray analysis. Differentially expressed genes were identified using linear models as implemented in the Bioconductor package LIMMA [32, 68]. Shown are the data for αIgM treated BL2 cells (triplicate) and from nine individual CD10^+^ tonsillar B cells stimulated *in vitro*. n.a. – not affected. BL2 data are part of the heatmap in Figure 1A and TABLE II, whereas gene expression changes of CD10^+^ tonsillar B cells are extracted from supplementary Table S3.

**Figure 2:**
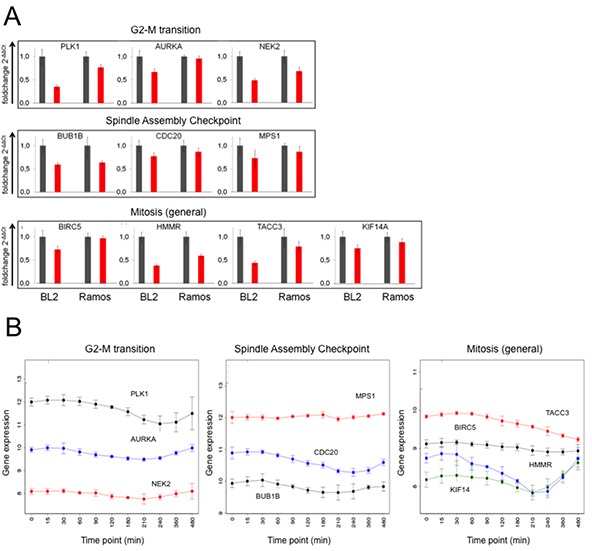
Expression of genes from the BCR.1 gene module is deliberately suppressed in lymphoma cells **A.** Expression of the genes for *PLK1, AURKA, NEK2, BUB1B, CDC20, MPS1, BIRC5, HMMR, TACC3* and *KIF14A* in response to αIgM treatment was analysed in BL2 and Ramos cells using qRT-PCR. One representative experiment out of three is shown. All samples were analysed in triplicate. Expression of the genes is shown as 2^−ΔΔCT^ relative to *abl* housekeeper expression and compared to unstimulated control. **B.** Time dependent suppression of genes as in Figure 2A by αIgM treatment of BL2 cells. Data are taken from Human ST1.0 microarray analysis. Differentially expressed genes were identified using linear models as implemented in the Bioconductor package LIMMA [68].

Of the 288 genes comprising the BCR.1 index *PLK1*, *AURKA*, *NEK2*, *BUB1B*, *CDC20, MPS1, BIRC5, HMMR*, *TACC3* and *KIF14A* were selected for validation and verification experiments by qRT-PCR in two independent cell lines [33–40]. These genes were selected because they are mitotic entry and progression regulating genes. BL2 and Ramos cells were stimulated with αIgM for 3hrs and gene expression changes analysed by qRT-PCR (Figure 2A). In line with our microarray data, qRT-PCR analyses show suppression of all selected BCR.1 genes by BCR activation. Furthermore, the expression of these genes was analysed over time following αIgM treatment (Figure 2B). Data were taken from a global gene expression analysis in BL2 cells. Cells were stimulated by αIgM and harvested at different time points starting from 30 min after adding αIgM until 480 min. RNA of three independent biological experiments was hybridized to Human ST1.0 microarrays. Most of the genes show a comparable time dependent suppression after αIgM treatment within the first 3-5hrs. Interestingly, some of the genes started to return to their basal expression level within 8hrs, suggesting a temporary effect on cell cycle regulation or corresponding negative feedback mechanism when cells are treated with αIgM (Figure 2B).

Next, we investigated whether BCR activation affects the same genes in lymphoma precursor cells in the same way as in cultured lymphoma cells. Therefore, the expression of cell cycle regulatory genes affected by αIgM treatment in BL2 cells was analysed in human tonsillar CD10^+^ B cells. Data were taken from a global gene expression analysis of CD10^+^ B cells from nine different donors (Table III, Supplementary Table S3). Although there are single BCR.1 genes that were not suppressed by BCR activation in human tonsillar CD10^+^ B cells, most of the analysed genes are suppressed by BCR stimulation as observed for BL2 cells. Thus, based on the common suppression of BCR.1 genes in cultured B lymphoma cells and in CD10^+^ human B cells, we conclude that this transcriptional regulation represents a common functional pathway of activated BCR signaling.

In addition, cell lines derived from DLBCL, U2932, HT, SUDHL5 and SUDHL6, were analysed for the presence of the BCR.1 index and whether it is affected by αIgM treatment. The BCR.1 index was detectable in HT and U2932 cells (Supplementary Figure S1A). Stimulation of DLBCL cell lines with αIgM led to a strong downregulation of BCR.1 genes (index increase) with the exception of SUDHL6 (Supplementary Figure S1B), indicating that down-regulation of BCR.1 index genes is also observed in DLBCL.

### A prolongation of G2/M transition in αIgM stimulated human B cells

**Figure 3:**
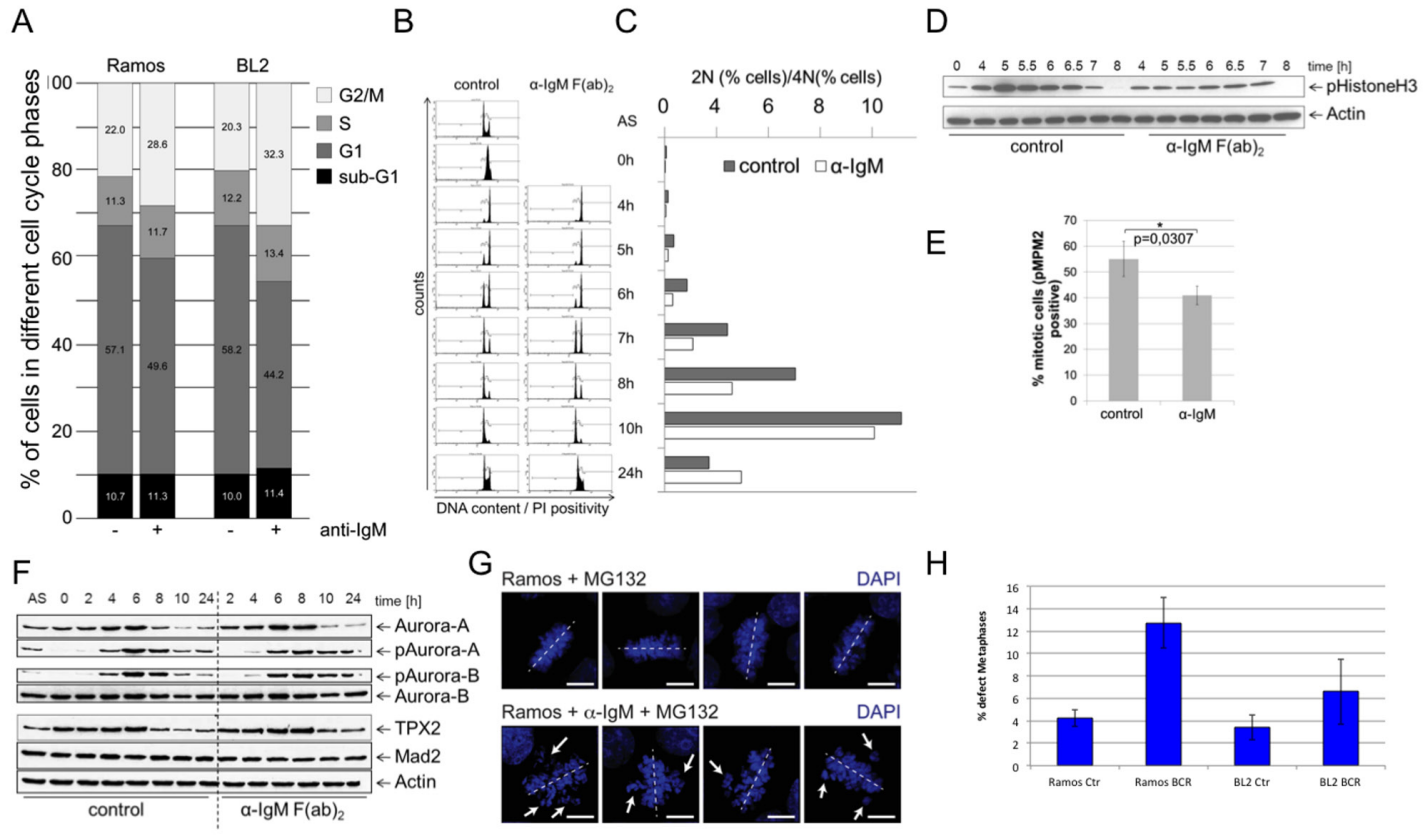
BCR activation is associated with a prolongation of the G2 phase, a deceleration of M phase entrance and metaphase defects **A.** Percent of BL2 and Ramos cells in different cell cycle phases with and without αIgM stimulation. Asynchronous growing BL cell line Ramos cells were stimulated by αIgM and cell cycle changes were detected using flow cytometry according to Nicoletti 6h after stimulation. **B.** Monitoring of the passage of synchronized Ramos cells through the cell cycle. Double thymidine block synchronized cells were released from cell cycle block and simultaneously stimulated using αIgM or left untreated. **C.** Percentage of cells within the different cell cycle phases as monitored by flow cytometry as in B. **D.** Changes in Histone 3 phosphorylation of synchronized Ramos cells after release from cell cycle block and simultaneously stimulated using αIgM or left untreated as monitored by immunoblot. **E.** Determination of the percentage of mitotic cells measured by phosphorylation of MPM2 (pMPM2) by flow cytometrical analyses of fixed cells 12h after stimulation. **F.** Thymidine synchronized Ramos cells were released from cell cycle block and treated as in B. Phosphorylation of AUROKA and AUROKB and their expression was monitored in unstimulated and αIgM stimulated cells in comparison to MAD2 and TPX2 as described recently [42]. Changes in Aurora kinase phosphorylation and TPX protein levels as measured by ImageJ quantification analysis shown within the Supplementary Figure S2. **G.** Detection of defective metaphases in αIgM stimulated cells quantified by microscopy. Thymidine synchronized Ramos cells were released from cell cycle block and treated as described in B. Two hours before harvesting cells were treated with 10μM MG132. Cells were stained with DAPI. **H.** The percentage of defect metaphases was calculated as monitored by microscopy exemplified in G.

Since many genes of the BCR.1 gene cluster are involved in the regulation of mitotic processes, we next studied if BCR signaling had an inhibitory effect on mitosis. Cell cycle progression was monitored using flow cytometry to define the percentage of the diploid and tetraploid sets of chromosomes [41, 42]. In asynchronously growing BL2 and Ramos cells, αIgM treatment led to a delay in G2/M as revealed by an increase in the percentage of cells with a 4N DNA content (Figure 3A). To obtain a better functional insight into the effects of BCR activation on cell cycle regulation, Ramos cells were synchronized at the G1/S transition. After release from thymidine block, cells where left untreated or stimulated by αIgM. Cell cycle progression was monitored using flow cytometry at defined time points as shown in Figure 3B. Within 4hrs both untreated or αIgM treated cells entered the G2 phase of the cell cycle. While untreated cells progressed through mitosis within 5-6hrs, αIgM treated cells were delayed by approximately 1-2hrs. Figure 3C shows the percentage of the 2N and 4N DNA content. Analyses of the phosphorylation of Histone 3 as a mitotic marker (pHistoneH3) showed that pHistone-H3 peaks after 5hrs in control cells in contrast to αIgM treated cells, which peaks at 6.5hrs (Figure 3D). After 8hrs no phosphorylation remains. Interestingly, the phosphorylation of this mitotic marker is generally decreased in cells activated via the BCR. To quantify the effect, the mitotic index of thymidine synchronized BL cells was analysed by measuring the percentage of pMPM3 positive cells. In αIgM treated cells the number of pMPM3 positive cells was lower compared to untreated cells (Figure 3E) further supporting the view that the activation of the BCR signaling pathway leads to a delay in G2 and/or lag in progression through the mitotic phase of the cell cycle. This is in agreement with the observed reduced expression of cell cycle regulators from the BCR.1 gene cluster. Immunoblot analysis for Aurora-A, Aurora-B, Tpx2 or Mad2 and the phosphorylated forms of Aurora-A and -B revealed corresponding changes in protein levels and phosphorylation patterns (Figure 3F). In line with a delay in mitotic progression, activation (phosphorylation) of the key mitotic kinases, Aurora-A and Aurora-B, was prolonged in BCR-activated BL cells (Figure 3F). Untreated cells enter M phase 4-6 hrs after release of the cell cycle block. After 8 hrs most of the cells have left mitosis as shown by a peak of TPX2 levels at 6hrs and the sharp decline of TPX2 after 8hrs (Figure 3F). However, cells that have been activated via BCR enter mitosis at the latest after 8hrs and need 10hrs to complete mitosis. The prolongation of the G2 phase as well as the observed delay of entry into mitosis could be an indication that BCR activation suppresses mitotic processes.

Therefore, defects in mitosis were investigated and quantified. Synchronized BL cells were released from cell cycle block as described above in the presence or absence of αIgM. 2hrs before harvesting, cells were treated with the proteasome inhibitor MG132 to arrest cells in metaphase. In Ramos cells treated with MG132 alone, metaphase chromosomes were found to be properly aligned at the equatorial plane. Abnormal metaphases were increased in αIgM treated cells (Figure 3H), suggesting that BCR signaling causes chromosome missegregation and subsequent accumulation of karyotype abberations.

**Figure 4:**
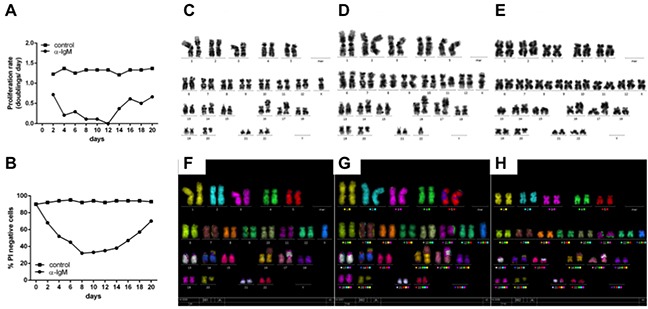
Continiously αIgM treated B cells are characterized by additional chromosomal aberrations **A.** Ramos cells were treated with αIgM or left untreated for at least 21 days adding αIgM every 24hrs after adjusting cell numbers according to untreated cells. Data are presented as proliferation rate (see additional details within the supplementary Material and Methods section). **B.** Cell viability of Ramos cells was measured by the number of propidium-iodide positive Ramos cells using FACS. **C.** Karyotype of Ramos cells in the absence of BCR activation. Karyotype of Ramos after chronic αIgM stimulation for 21 **D.** and 28 days **E.** respectively. **F.** M-FISH of Ramos cells in the absence of BCR activation. M-FISH of Ramos cells after chronic αIgM stimulation for 21 **G.** and 28 days **H.** respectively. Shown are additional chromosome aberrations of add(16)(q24) after 21d (**D/G**) and of t(15;21)(q14?;q22) after 28days (**E/H**) αIgM treatment respectively.

To test this, a chronic activation of BCR was mimicked by culturing Ramos cells over 3 weeks with daily addition of αIgM F(ab)_2_ fragments. In Figure 4 A/B cell viability and cell doubling data are presented from these experiments. A decrease of cell viability within the first 10 days was accompanied by a very low cell doubling. This fits well with earlier observations of increased cell death in BL cells after *in vitro* BCR activation monitored as cell cycle arrest within G1 [43]. However, after two weeks both cell proliferation and viability were increased. Analysis of the karyotypes of chronically treated Ramos cells revealed additional chromosomal aberrations including translocations (t(15;21)(q14?;q22)) as well as additions (add(16)(q24)) (Figure 4). These structural abnormalities were not observed in untreated BL cells. One explanation for the induction of additional chromosomal abberations could be that chronic exposure of B cells to antigens and thus BCR activation can lead to subsequent cell cycle changes predisposing cells for the acquisition of subsequent chromosomal aberrations.

### Gene suppression is negatively associated with c-Myc activity after B cell receptor activation

It was shown recently that in BL cell lines αIgM treatment is associated with a PI3K dependent decrease in *MYC* expression [32]. Therefore, the involvement of c-Myc in BCR.1 gene regulation, particularly on cell cycle regulators and on cell cycle deregulation induced by αIgM was evaluated.

**Figure 5:**
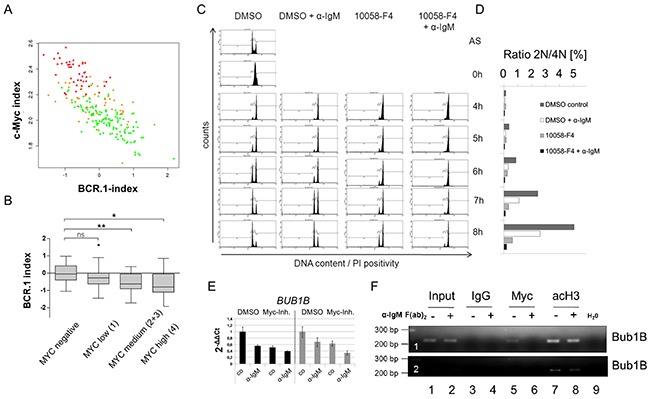
c-Myc is involved in the regulation of cell cycle regulators from the BCR.1 gene cluster **A.** The BCR.1 index is inversely correlated with the c-Myc index in distinct groups of lymphoma patients and discriminates Burkitt lymphoma from diffuse large B cell lymphoma. The parallel activity was estimated plotting the BCR.1 and c-Myc indices against each other and calculating the respective correlation coefficient. The correlation coefficients of the BCR.1 index and the c-Myc index were calculated in gene expression profiles of 219 aggressive NHL [30]. The NHL cases were assigned to the following molecular categories: mBL (red), non-mBL (green) and intermediate lymphoma (yellow) based on their gene expression profiles [30]. **B.** The BCR.1 index is inversely correlated to the number of c-Myc positive cells in NHL. c-Myc protein levels were determined using immunohistochemical staining of tissue-microarrays. The scoring was performed as follows: low expression (0-25% positive cells) and high expression (25-100 % positive cells). **C.** B cell receptor activation and c-Myc-inhibition delays the G2/M cell cycle phase transition in an additive way as monitored by the passage of synchronized Ramos cells through the cell cycle. Double thymidine block synchronized Ramos cells were analysed as described in Figure 3. **D.** Percentage of cells within the different cell cycle phases monitored by flow cytometry as in C. **E.** qRT-PCR analysis of *BUB1B* gene expression in BL2 (black bars) or Ramos cells (grey bars). BL2 and Ramos cells were pretreated for 3h with 60μM 10058-F4 c-Myc inhibitor or solvent (DMSO). Cells were stimulated for an additional 3h with αIgM F(ab)2 fragment (12μg/ml). qRT-PCR analyses were performed using SYBR green. Fold changes were calculated using the ΔΔCt method. One representative experiment of three replicates is shown. Additional BCR.1 genes are shown in the extended view figure E1. **F.** c-Myc binds to the *BUB1B* gene. A fragment was amplified that encompasses the previously described E-box in intron 1 of the *BUB1B* gene [45]. ChIP was performed using antibodies directed against IgG as a negative control (lanes 3,4), against c-Myc (lanes 5,6) and against acetylated histone H3 as positive control (marker for active transcription) (lanes 7,8). c-Myc binds to the *BUB1B* gene (lane 5) but this binding is lost as a result of B cell receptor activation (lane 6). The lower electropherogram shows a shorter exposure time to show differences in acetyl Histone H3 binding.

First, a patient-based comparison between the BCR.1 index with our recently established Myc-index was performed [20]. The correlation coefficients of these two indices were calculated in gene expression profiles of 219 B-NHL including molecularly defined BLs (mBLs), non-mBLs and intermediate lymphoma (molecularly unclassified B-NHLs) [30]. The correlation coefficient is −0.76 (Figure 5A). Thus, the BCR.1 index inversely correlates with the c-Myc index. Figure 5A shows both index values per sample. Therefore, mBL cases are characterized by a high c-Myc but a low BCR.1 index, whereas non-mBLs and intermediate lymphoma are more heterogeneous with a lower c-Myc and higher BCR.1 index. Lymphomas with a high BCR.1 signal can be characterized by a very low c-Myc activity and therefore can be grouped as BCR.1^high^/Myc^low^ NHL. The majority of non-mBLs and intermediate lymphoma are characterized by corresponding higher BCR.1 but lower c-Myc activity relative to mBLs. Comparison of the BCR.1 index with BCL6 regulated genes was performed in patient samples (Supplementary Figure S3) [28, 44]. Although a good separation of mBLs from intermediate and non-mBLs was observed, the index correlation (−0.1) was not as strong as for c-Myc. Therefore, the c-Myc protein levels were scored on tissue microarrays (TMA) of 99 lymphoma cases taken from the MMML-cohort described above [30]. In parallel, the BCR.1 index for these cases based on their gene expression profiles was calculated. This analysis revealed that cases with higher numbers of c-Myc positive lymphoma cells were significantly more likely to express BCR.1 genes (low BCR.1 index) (p=0.0012) (Figure 5B). This finding underscores the negative correlation of c-Myc and BCR.1 indices and supports the definition of a BCR.1^high^ Myc^low^ NHL subgroup (mainly DLBCL).

To assess whether the cell cycle delay after BCR activation might be the result of the BCR induced inhibition of c-Myc, we investigated whether the inhibition of c-Myc alone can induce G2-phase prolongation of thymidine synchronized Ramos cells. Double thymidine block synchronized Ramos cells were released from cell cycle block and treated with the chemical Myc inhibitor 10058-F4, αIgM, or both. Within 4hrs all cells entered the G2 phase of the cell cycle. While untreated cells entered the G1 phase of the cell cycle within 5-6hrs αIgM, 10058-F4 and αIgM/10058-F4 treated cells were delayed (Figure 5C). The prolonged G2 phase in 10058-F4 treated cells was comparable to the effect of αIgM treatment alone and thus supports the inverse correlation of BCR.1 and the Myc index shown in Figure 5A/B. The strongest delay was observed by the combination of BCR activation and c-Myc inhibition. Therefore, αIgM treatment together with 10058-F4 shows an additive effect on G2 phase prolongation. This suggests that the BCR effect on the cell cycle regulation is not solely the result of inhibiting *MYC* expression. In Figure 5D the percentage of the diploid and tetraploid sets of chromosomes is shown.

The role of aberrant c-Myc activity on cell cycle and BCR.1 gene regulation was analysed by qRT-PCR as shown for *BUB1B* and other BCR.1 genes in BL2 and Ramos cells treated with 10058-F4, αIgM or both (Figure 5E, Supplementary Figure S4). The expression of some BCR.1 genes is dependent on aberrant c-Myc activity, but BCR activation and c-Myc inhibition act in an additive manner.

In addition we tested by chromatin-immunoprecipitation (ChIP) whether c-Myc binding to a known E-box containing region in the first intron of the *BUB1B* gene is altered upon BCR activation [45, 46]. The gene locus of the *BUB1B* gene was enriched by ChIP of c-Myc in unstimulated cells (Figure 5F). Importantly, following BCR activation this enrichment was abolished. As positive control a ChIP of acetylated histone H3 was used as a marker for active transcription. Therefore, a critical cell cycle regulator is suppressed in response to suppression of *MYC* gene expression by αIgM. However, the observed additive effect of BCR activation together with 10058-F4 treatment also indicates that additional pathways are involved through BCR activation which could mediate the suppression of the BCR.1 genes.

### The BCR.1 gene module characterizes individual NHL and is more active within GCB-like DLBCL

**Figure 6:**
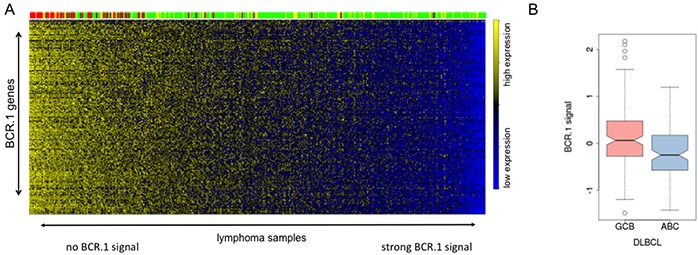
The BCR.1 index characterizes individual aggressive NHL and discriminates between ABC- and GCB-like DLBCL **A.** The heatmap is showing the expression of BCR.1 genes (row) in molecular profiles of 389 primary lymphoms samples (columns) from distinct patient cohorts [30, 47]. The patient samples are ordered according to rising BCR.1 index from left to right. The colour-coded bar on top of the heatmaps represents the affiliation of patients tomBL (red), non-mBL (green) and yellow (intermediate) diagnosis. **B.** Boxplot of the distribution of indices for BCR-repressed genes comparing 281 DLBCL samples based on their relation to GCB- and ABC-like DLBCL classification. The difference is highly significant with p=9.35e−06.

To further study the functional relevance of the BCR.1 gene module in specific subgroups of B-NHL and specifically in DLBCL, we compared by the gene expression profiles of 389 primary lymphoma samples [30, 47]. Figure 6A shows that mBLs (colored in red) are characterized by a low or absent BCR.1 index. This was also observed for samples characterized by *MYC*-aberrations. About 20% of analysed non-mBLs show a strong coherently low expression of BCR.1 genes and therefore are characterized by a strong corresponding BCR signal. This is in good agreement with the observation presented in Figure 1B within the “training cohort” and the observed inverse correlation of the BCR.1 and c-Myc indices (Figure 5A). A comparison of ABC- and GCB-like DLBCLs in this analysis is difficult as mBLs are labelled also as GCB-like lymphomas. Therefore, an additional analysis was performed (Figure 6B). Comparing 281 non-mBL DLBCL samples, we observed a significantly higher BCR.1 index in GCB-DLBCL (n=159) compared with ABC-like DLBCL (n=122) (p=9.35e−06). This supports the view that more GCB-like DLCBL are characterized by a specific coexpression of BCR.1 genes than are ABC-like DLBCL.

## DISCUSSION

Gene expression based molecular tumour signatures have been identified in aggressive NHL. In DLBCL specific oncogenic signalling networks have been deduced from these signatures. These networks are driven by mutations or the microenvironment acting onto the lymphoma cells [1, 13, 48, 49]. However, the functional effects revealed by these gene expression signatures, are poorly understood. A better understanding could improve of lymphoma treatments. Therefore, the present study aimed to gain further functional insights into oncogenic pathways active in aggressive NHL. The guided clustering approach was extended to identify transcriptional modules that are conserved between patients and *in vitro* stimulated cells but dominantly activated or inhibited by for example BCR signaling but not by other well known mediators of signalling in B cells. We have provided a comprehensive genome-wide resource for the functional exploration of molecular and cellular regulation in B cells. Ten new transcriptional modules were identified (Figure 1).

Of the ten transcriptional modules we identified, the BCR.1 module comprises genes, which are suppressed by BCR activation. It was shown that these genes are downregulated by BCR activation in a number of different cell lines including BL2, Ramos, U2932, HT and SUDHL5. We conclude that in patients BCR or related pathways are involved in the described way of gene coregulation. Furthermore, the BCR.1 module is strong in a substantial number of GCB-like DLBCL but not in mBL and supports the hypothesis, that in some DLBCL BCR signaling is involved in the downregulation of cell cycle regulators. Because in ABC-like DLBCLs the BCR.1 signal is attenuated compared to GCB-like DLBCLs (Figure 6B), it would be interesting to determine if the coactivity of classical Jak/STAT and NF-κB signaling characteristic of ABC-like DLBCL can enhance the BCR.1 gene expression independent of c-Myc [19, 50].

Remarkably, BCR.1 genes are enriched for those involved in cell cycle regulation (Figure 2 and Table III). The functional outcome of this gene module is a delayed entry to and progression of mitosis most probably as a result of reduced expression of the identified cell cycle regulators (Figure 3). After continuous BCR activation some B cells gain additional genetic aberrations (Figure 4) as a direct or indirect consequence of the observed changes in G2/M cell cycle phase delays. By large-scale follow-up analyses of pairs of primary and relapsed aggressive NHL it can now be tested which role BCR.1 gene expression is playing to affect increases of chromosomal aberrations or disease progression. However, this is also an important observation as the genes suppressed by BCR activation are also found to be downregulated in isolated human tonsillar CD10^+^ B cells. Therefore, it is proposed that these genes are functionally relevant in both transformed and in reactive B cells.

There is growing evidence of a role for BCR signaling in the pathogenesis of different subtypes of NHL. In BL a tonic BCR signal is observed whereas in DLBCL both tonic and chronic active BCR signaling seem to be important [26, 27, 51–53]. As well as supporting B cell proliferation, survival or differentiation, BCR activation can also induce G1 cell cycle arrest, apoptosis and increased cell death in different B cells [54–59]. Our observations suggest that these suicide pathways can be, at least partially, attributed to a delay in G2/M with associated mitotic defects potentially leading to subsequent chromosomal aberrations. Our data also provide a plausible mechanism to explain the link between inflammation and the development of lymphomagenic mutations; especially in DLBCL with a high BCR.1, chronic inflammation and/or chronic antigen driven activation of oncogenic pathways with no or low c-Myc activity. The inverse correlation of the Myc-index and the BCR.1 index suggests that in lymphomas with a high *MYC* expression corresponding BCR.1 genes are highly expressed (low BCR.1 index, high gene expression) and thus most likely indicates an absence of a strong antigen stimulation.

The newly identified oncogenic network present in a substantial number of GCB-like DLBCL will likely define more rational treatment targets in this heterogeneous disease. Whether this has implications for stratifying NHL patients for molecular-based prognostication and for targeted therapy has to be investigated in new clinical trials using R-CHOP and/or currently introduced targeted drugs for Btk, PI3K and NF-kB pathways or BET-domain containing protein inhibitors [25, 48, 51, 60].

## MATERIALS AND METHODS

### Cell culture

BL2, Ramos, SUDHL5, SUDHL6, U2932 and HT (all obtained from the DSMZ Braunschweig, Germany) cell cultivation as well as purification and sorting of primary human tonsillar B cells was performed as previously described [20, 61] [32]. A detailed protocol can be found in the “Extended View Methods Section”. For stimulation studies, cells were cultured as described previously [32]. Cells chronically activated by αIgM were grown for up to 28 days and αIgM was added every 24hrs. Cells were harvested using corresponding inhibitors of phosphatases and proteases and RNA isolated using the RNeasy Plus Mini Kit (Qiagen). Cell doubling and viability was determined by counting cells in the presence of trypan blue.

Synchronisation of BL cells was performed using thymidine treatment as recently described [62]. Cell cycle analysis was performed on the basis of analysing the DNA content in the nuclei of the cells by propidium iodide staining followed by flow cytometry. For a detailed description see the “Expanded View Methods Section”.

### Gene expression analysis

qRT-PCR was performed using SYBR green. ΔCt values were normalised to ß2m and abl expression and ΔΔCt values calculated. Oligonucleotides used are summarized in Supplementary Table S4. For whole genome microarrays Human Genome U133A 2.0 plus Arrays (Affymetrix) or Human ST1.0 Arrays (Affymetrix) as indicated was performed according to manufacturer's recommendations by the TAL (UMG, Germany) For further details of gene expression analyses see the “Expanded View Methods Section”.

### Cytogenetic analysis

Metaphases were analysed using chromosome banding analysis and 24-color FISH. Chromosome preparation was performed using standard cytogenetic protocols and modified chromosome banding technology (GAG// Giemsa bands of acetic Saline Giemsa). M-FISH (24 color FISH) was done according to the manufacturer's protocol (MetaSystems). Karyotypes were classified according to the International System for Human Cytogenetic Nomenclature (ISCN) [63].

### Chromatin immunoprecipitation

BL2 cells were stimulated with 1.3μg/ml αIgM F(ab)_2_ fragments for 3hrs or left untreated as control. Cells were sedimented and resuspended in PBS containing 1.42% formaldehyde and incubated for 15min at room temperature. Chromatin-Immunoprecipitation was conducted as described previously using 2μg anti c-Myc (clone N-262, Santacruz), 2μg anti acH3 (clone 06-599, Millipore), 2μg αIgG control (ab46540, abcam) antibodies. A detailed protocol is presented in the “supplemental Material and Methods section”.

### Case selection and classification for immunohistochemistry

127 B-cell lymphoma samples were obtained from the files of the lymph node registry Kiel with ethical approval and classified according to the WHO classification using standard histological, immunohistochemical and molecular criteria [64]. In addition, 20 specimens obtained within the framework of the network project “Molecular Mechanisms in malignant Lymphoma” (MMML) were analysed. The latter cohort was diagnosed and classified based on the previously reported molecular signatures [30]. All lymphomas were analysed using tissue-micro-arrays (TMA) containing 2 cores of 1 mm or 0.6 mm (for the MMML specimen) for each case (ethical approval Ref. Nr. 1/1/05).

### Bioinformatic analyses and analysed datasets

The identification of pathway activation clusters was performed using the Guided Clustering algorithm [28]. Guided Clustering identifies genes specific for one of the five stromal stimuli (BCR, CD40L, BAFF, IL-21, LPS). Within this framework the stimulated BL2 cells act as guiding data whereas the cohort of Myc-negative DLBCL represent the guided dataset [30]. The definition of the required binary vector depends on the respective stimulation with the numerical value 1 for the stimulus and 0 for the remaining stimuli as well as for the controls. For each stimulation two clusters were extracted, showing an opposite regulation. The respective genes in each cluster may represent a potential surrogate marker for pathway activity in lymphoma samples. To determine the extent of gene cluster activity in lymphoma patients, one index was calculated by gene cluster and per lymphoma sample. Expression values from those genes assembling the individual cluster were used to calculate a single representative value (the index) for each sample by fitting a standard additive model with independent gene and sample effects using Tukey's median polish procedure as described [65]. The index values of genes inhibited by a stimulus (.1 modules (eg BCR.1)) were multiplied by minus one to enable the interpretation of the indices as absence or presence of stimulation. Hence, down-regulation of these genes leads to a high index value (stimulation is present) whereas a low index value indicates the absence of any regulatory effect due to stimulation. Gene set enrichment analysis (GSEA) of ranked gene list was performed using the Java implementation of GSEA obtained from http://www.broadinstitute.org/gsea/. GSEA was conducted in the mode for pre-ranked gene lists on the C2 set of curated gene signatures from the Molecular Signature Database (MSigDB). Genes are attributed to one ore more biological processes, molecular function and cellular location with respect to the Gene Ontology as well as pathways in the KEGG database [66, 67]. Hypergeometric testing was performed to test for non-random overrepresentation of module genes in each of these terms based on the hypergeometric distribution model. This GO/KEGG analyses was implemented in customized inhouse scripts.

The primary data are available from GEO (http://www.ncbi.nlm.nih.gov/geo/) - raw data for gene expression changes for LPS, CD40L, BAFF, IL-21 and αIgM stimulated BL2 cells are available under accession no. GSE42660 and GSE29700 [28, 32], data for CD10^+^ human tonsillar B cells (GSE71724), pathway inhibition (GSE68761) and gene expression kinetics (GSE71721) are summarized as SuperSeries record GSE71725.

## SUPPLEMENTARY METHODS FIGURES AND TABLES










