# Supplementary material for: Identification of a new gene regulatory circuit involving B cell receptor activated signaling using a combined analysis of experimental, clinical and global gene expression data
Source: Oncotarget. 2016 May 7;7(30):47061–81. doi: 10.18632/oncotarget.9219 (PMC5216924; doi:10.18632/oncotarget.9219)
Supplement: Supplementary file 1 [file oncotarget-07-47061-s001.pdf]

SUPPLEMENTARY METHODS

Cell culture of chronically αIgM stimulated BL2 cells

Cells chronically activated by αIgM were grown for up to 28 days and αIgM was added every 24hrs. Cell numbers were determined every two days by counting cells in the presence of trypan blue and cells were seeded in fresh media. Proliferation rates were calculated as described by Sullivan et al. (Sullivan et al, 2015):

Proliferation rate (doublings/day) = $\log_2$ (cells/ml (day x)/ cells/ml (day x-2))/2 days.

Purification and sorting of primary human tonsillar B cells

Tonsils were taken from pediatric patients with informed consent and with ethical approval (Ref No.06/Q2702/50). Mononuclear cells from tonsils were isolated and stimulated and sorted as described previously (Schrader et al, 2012; Vockerodt et al, 2008; Vockerodt et al, 2001). For stimulation studies, cells were cultured in cell culture medium supplemented with 10mM HEPES at $1x10^6$ cells/ml and incubated with indicated reagents for up to 9hrs. To crosslink the BCR, cells were cultured in the presence of 1.3µg/ml goat α-IgM $F(ab)_2$ fragments (Jackson Immunity).

Gene expression analysis

Gene expression analysis via qRT-PCR was conducted via SYBR green based realtime PCR analyses. For whole genome micorarrays Human Genome U133A 2.0 plus Arrays (Affymetrix) or Human ST1.0 Arrays (Affymetrix) as indicated was performed according to manufacturer's recommendations by the TAL (UMG, Germany). For gene expression analysis RNA was isolated with RNeasy Plus Mini Kit (Qiagen) according to the manufacturer's instructions. For real time PCR analysis RNA was reverse transcribed using SuperScript II Reverse Transcriptase (Invitrogen) and random hexamer primers (IBA BioTAGnology). cDNA samples were further analysed by SYBR Green-based real-time PCR using the 7900HT Fast Real-Time PCR System (Applied Biosystems) (additional details for used primers within the Table E4). For whole genome micorarrays RNA was labelled for microarray hybridization using Affymetrix GeneChip® IVT Labelling Kit (Affymetrix). Fragmentation and hybridization of labelled antisense RNA on Human Genome U133A 2.0 plus Arrays (Affymetrix) or Human ST1.0 Arrays (Affymetrix) as indicated were processed according to manufacturer's recommendations by the TAL (UMG, Germany). Gene expression values were obtained by first correcting for the background and normalizing on probe level using the variance stabilization method by Huber and colleagues (Huber et al, 2002). The normalized probe intensities were summarized into gene expression levels by using an additive model fitted by the median polish procedure (Hoaglin, 1977; Irizarry et al, 2003).

Cell cycle analysis and definition of the mitotic index

Cell cycle analysis was performed on the basis of analysing the DNA content in the nuclei of the cells by propidium iodide staining and followed by flow cytometric analyses. Therefore, an 200µl of the cell suspension was sedimented (250 x g, 10min, RT) and the pellet was resuspended in 500µl hypotonic Nicoletti solution (Nicoletti et al, 1991). This buffer leads to the destruction of the cell membrane due to hypotonic stress, whereas the nucleus stays intact. Propidium iodide respectively DNA content of the nuclei was determined by flow cytometry. Distribution of the cells according to the phase of the cell cycle was performed with the help of FlowJo™ (Treestar, Ashland USA) software according to Dean-Jett-Fox (Fox, 1980) and by CellQuest™ (Becton Dickinson, Oxford UK) software. The nuclei of viable cells show three characteristic distributions in the flow cytometric analysis based on their differing DNA content. By this analysis method one can discriminate the cell cycle phases G1, S und G2. Furthermore nuclei from apoptotic cells show an even smaller amount of DNA as cells in the G1 phase and are therefore summarized in the group of SubG1 events.

Chromatin immunoprecipitation

BL2 cells were stimulated with 1.3µg/ml a-IgM $F(ab)_2$ fragments for 3hrs or left untreated as control. Cells were sedimented and resuspended in PBS containing 1.42% formaldehyde and incubated for 15min at room temperature. Chromatin-Immunoprecipitation was conducted as described previously (Nagarajan et al, 2014). The formaldehyde was quenched by adding 1ml 1.25M Glycine. Cells were washed withice-cold PBS and resuspended in IP-buffer containing a proteinase inhibitor cocktail (Complete Roche). The nuclear pellet was washed with IP-buffer. Nuclei in IP buffer were sonicated using the Biorupter device from Diagnode. Protein A Sepharose was blocked by adding 15ml of IP buffer containing BSA and salmon sperm DNA. The swollen sepharose was washed with IP-buffer. 1 volume of IP buffer was added to the sepharose. For preclearing 80 µl blocked sepharose was added to sheared and diluted chromatin and incubated for 1h at 4°C. Sepharose

was sedimented. The precleared lysates were used for IP at 4°C overnight (2µg anti c-Myc (clone N-262, Santa Cruz), 2µg anti acH3 (clone 06-599, Millipore), 2µg anti IgG control (ab46540, abcam) and Input control). Protein A - sepharose was used for antibody precipitation. To release DNA from protein immune complexes Chelex 100 slurry was added to washed beads and to the precipitated Input DNA. Samples were mixed and heated to 95°C for 10min. After boiling proteinase K was added to the cooled samples. Incubation was performed at 55°C for 30 min.

REFERENCES

Fox MH (1980) A model for the computer analysis of synchronous DNA distributions obtained by flow cytometry. *Cytometry* 1: 71-77.

Hoaglin DCeae (1977) Understanding Robust and Exploratory Data Analysis. *John Wiley & Sons, New York, USA*.

Huber W, von Heydebreck A, Sultmann H, Poustka A, Vingron M (2002) Variance stabilization applied to microarray data calibration and to the quantification of differential expression. *Bioinformatics* 18: S96-104.

Irizarry RA, Ooi SL, Wu Z, Boeke JD (2003) Use of mixture models in a microarray-based screening procedure for detecting differentially represented yeast mutants. *Stat Appl Genet Mol Biol* 2: Article1.

Nagarajan S, Hossan T, Alawi M, Najafova Z, Indenbirken D, Bedi U, Taipaleenmaki H, Ben-Batalla I, Scheller M, Loges S, Knapp S, Hesse E, Chiang CM, Grundhoff A, Johnsen SA (2014) Bromodomain protein BRD4 is required for estrogen receptor-dependent enhancer activation and gene transcription. *Cell Rep* 8: 460-469.

Nicoletti I, Migliorati G, Pagliacci MC, Grignani F, Riccardi C (1991) A rapid and simple method for measuring thymocyte apoptosis by propidium iodide staining and flow cytometry. *J Immunol Methods* 139: 271-279.

Schrader A, Bentink S, Spang R, Lenze D, Hummel M, Kuo M, Arrand JR, Murray PG, Trumper L, Kube D, Vockerodt M (2012) High myc activity is an independent negative prognostic factor for diffuse large B cell lymphomas. *Int J Cancer* 131: E348-361.

Sullivan LB, Gui DY, Hosios AM, Bush LN, Freinkman E, Vander Heiden MG (2015) Supporting Aspartate Biosynthesis Is an Essential Function of Respiration in Proliferating Cells. *Cell* 162: 552-563.

Vockerodt M, Morgan SL, Kuo M, Wei W, Chukwuma MB, Arrand JR, Kube D, Gordon J, Young LS, Woodman CB, Murray PG (2008) The Epstein-Barr virus oncoprotein, latent membrane protein-1, reprograms germinal centre B cells towards a Hodgkin's Reed-Sternberg-like phenotype. *J Pathol* 216: 83-92.

Vockerodt M, Tesch H, Kube D (2001) Epstein-Barr virus latent membrane protein-1 activates CD25 expression in lymphoma cells involving the NFkappaB pathway. *Genes Immun* 2: 433-441.

SUPPLEMENTARY FIGURES AND TABLES

A)

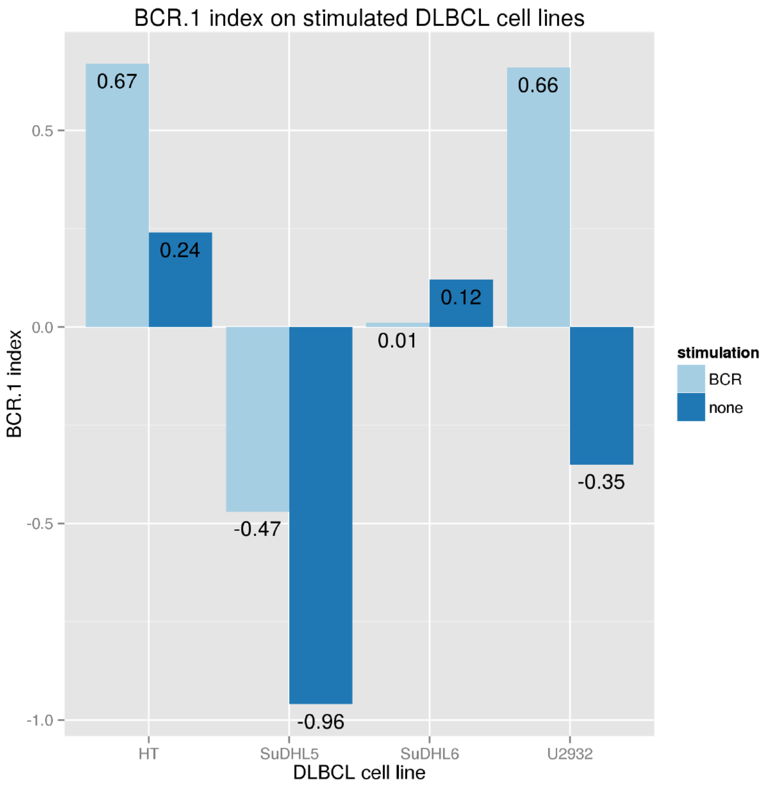

B)

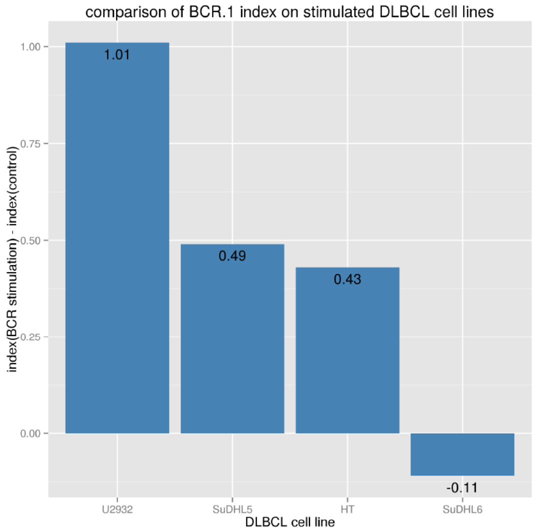

Supplementary Figure S1: The BCR.1 index in DLBCL cells. RNA from unstimulated and αIgM treated SUDHL5, SUDHL6, HT and U2932 cells was isolated and hybridized on Human Genome U133A 2.0 plus Arrays. BCR.1 genes were analysed as for BL2 cells.

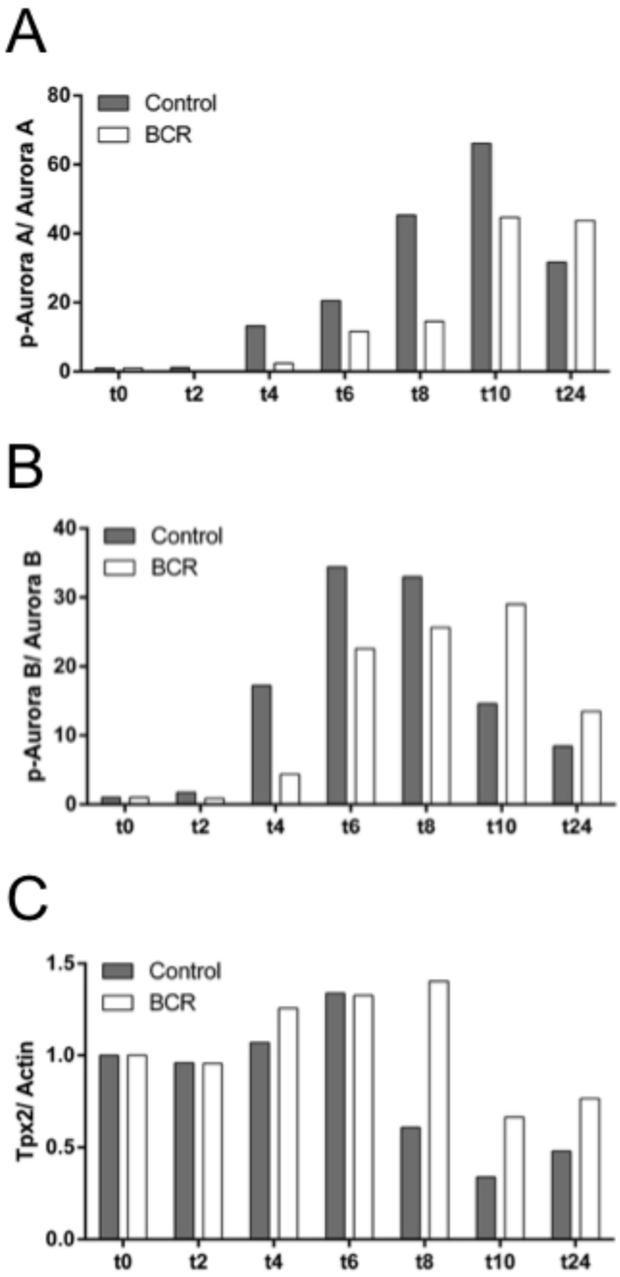

Supplementary Figure S2: BCR activation is associated with a delay in G2/M cell cycle phase. Thymidine synchronized Ramos cells were released from cell cycle block and treated as in described in Figure 3B. Phosphorylation of AUROKA and AUROKB and their expression was monitored in unstimulated and αIgM stimulated cells in comparison to MAD2 and TPX2 as described recently [42]. Changes in Aurora kinase phosphorylation and TPX protein levels are quantified by ImageJ.

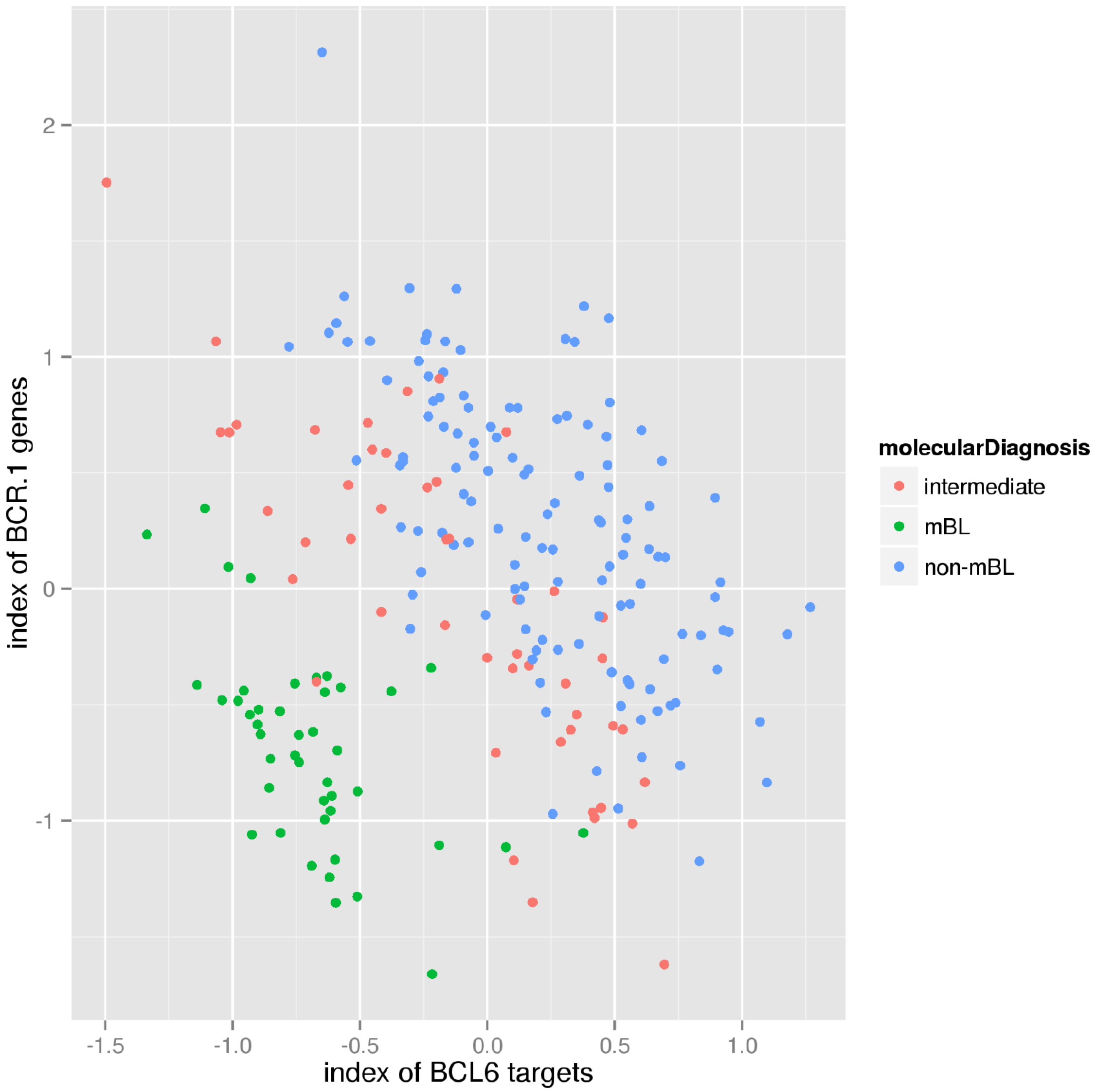

Supplementary Figure S3: Index comparison of the BCR.1 index vs BCL6 targtet genes in distinct groups of lymphoma patients and discriminates Burkitt lymphoma from diffuse large B cell lymphoma. The parallel activity was estimated plotting the BCR.1 index and BCL6 target genes against each other and calculating the respective correlation coefficient (index correlation -0.1). The correlation coefficients of the BCR.1 index and the BCL6 target gene index were calculated in gene expression profiles of 219 aggressive NHL [30]. The NHL cases were assigned to the following molecular categories: mBL (red), non-mBL (green) and intermediate lymphoma (yellow) based on their gene expression profiles [30]. The BCL6 target genes are defined as described before [28, 44].

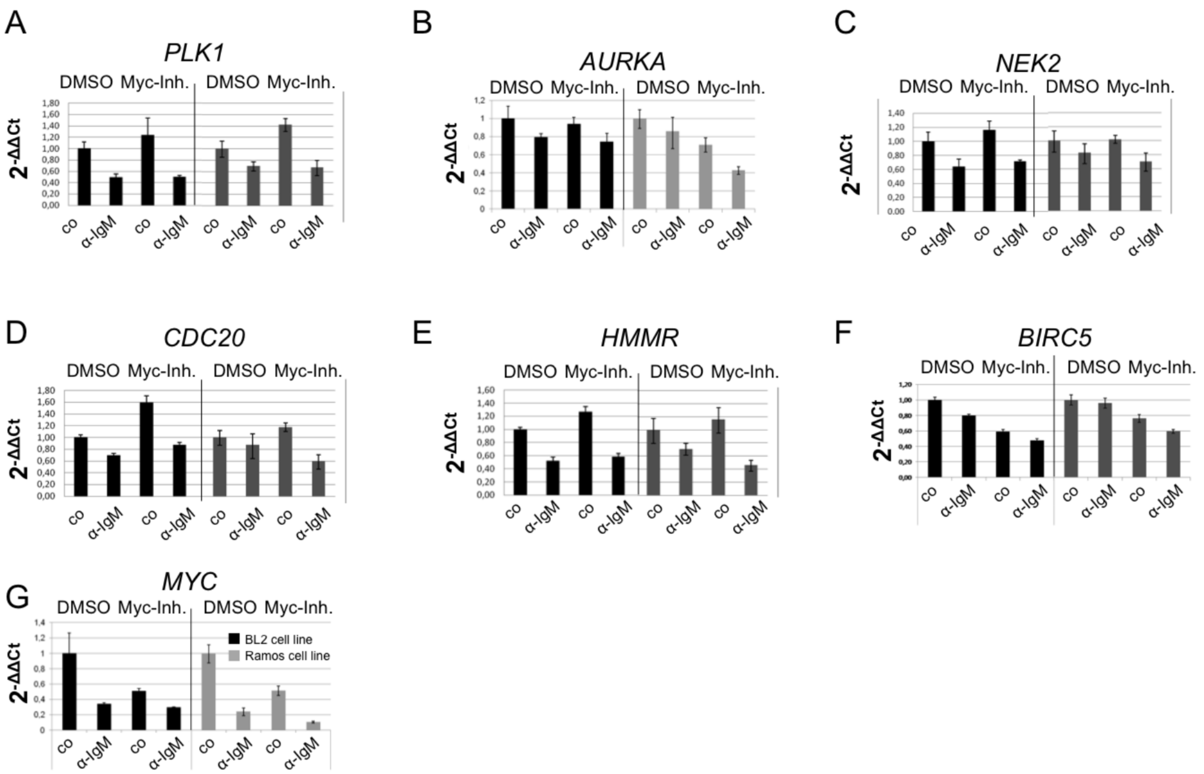

Supplementary Figure S4: Gene expression of *PLK1, AURKA, NEK2, CDC20, BIRC5, HMMR* in comparison to *MYC* after treating cells with 10058-F4, αIgM or both. qRT-PCR analysis of gene expression in BL2 (black bars) or Ramos cells (grey bars). BL2 and Ramos cells were pretreated for 3h with 60µM 10058-F4 c-Myc inhibitor or DMSO (co). Cells were stimulated for additional 3h with αIgM F(ab)2 fragment (12µg/ml). qRT-PCR analyses were performed using SYBR green. Fold changes were calculated using the ΔΔCt method. One representative experiment of three replicates is shown.

Supplementary Table S1: Coherently in lymphoma expressed genes dominantly affected by *in vitro* interventions of one stimuli in comparison to the other stimuli used. Identification of genes building different clusters of genes displaying a coherent expression across patient profiles affected by multiple *in vitro* interventions using guided clustering.

See Supplementary File 1

Supplementary Table S2: Gene Set Enrichment Analyses (GO). Gene Set Enrichment for BCR.1 genes was performed using the Geneset annotations implemented in the GO database. The TOP100 most significantly enriched genes sets corresponding to biological processes are shown below.

See Supplementary File 2

Supplementary Table S3: Gene expression changes in primary human $CD10^+$ B cells after B cell receptor crosslink: Differentially expressed genes were identified using linear models as implemented in the Bioconductor package LIMMA [68]. False discovery rates for lists of differentially expressed genes were calculated according to Benjamini and Hochberg in a paired-test as described in the Material and Methods section.

See Supplementary File 3

Supplementary Table S4: Oligonucleotides used in the described experiments.

See Supplementary File 4
