# Supplementary material for: Identification of a new gene regulatory circuit involving B cell receptor activated signaling using a combined analysis of experimental, clinical and global gene expression data
Source: Oncotarget. 2016 May 7;7(30):47061–81. doi: 10.18632/oncotarget.9219 (PMC5216924; doi:10.18632/oncotarget.9219)
Supplement: Supplementary file 3 [file oncotarget-07-47061-s003.docx]

**Table E2** **Gene Set Enrichment Analyses (GO)** Gene Set Enrichment for BCR.1 genes was performed using the Geneset annotations implemented in the GO database. The TOP100 most significantly enriched genes sets corresponding to biological processes are shown below

| **Biological Process** | | | |
| --- | --- | --- | --- |
|  | **GO ID** | **GO Term** | **downreg. p-value** |
| **1** | [GO:0007017](http://amigo.geneontology.org/cgi-bin/amigo/term-details.cgi?term=GO:0007017) | microtubule-based process | 6,00E-16 |
| **2** | [GO:0000278](http://amigo.geneontology.org/cgi-bin/amigo/term-details.cgi?term=GO:0000278) | mitotic cell cycle | 7,00E-15 |
| **3** | [GO:0006996](http://amigo.geneontology.org/cgi-bin/amigo/term-details.cgi?term=GO:0006996) | organelle organization | 5,00E-14 |
| **4** | [GO:0000226](http://amigo.geneontology.org/cgi-bin/amigo/term-details.cgi?term=GO:0000226) | microtubule cytoskeleton organization | 1,00E-13 |
| **5** | [GO:0051301](http://amigo.geneontology.org/cgi-bin/amigo/term-details.cgi?term=GO:0051301) | cell division | 2,00E-13 |
| **6** | [GO:0007059](http://amigo.geneontology.org/cgi-bin/amigo/term-details.cgi?term=GO:0007059) | chromosome segregation | 3,00E-13 |
| **7** | [GO:0006281](http://amigo.geneontology.org/cgi-bin/amigo/term-details.cgi?term=GO:0006281) | DNA repair | 5,00E-12 |
| **8** | [GO:0006974](http://amigo.geneontology.org/cgi-bin/amigo/term-details.cgi?term=GO:0006974) | response to DNA damage stimulus | 2,00E-11 |
| **9** | [GO:0007051](http://amigo.geneontology.org/cgi-bin/amigo/term-details.cgi?term=GO:0007051) | spindle organization | 4,00E-09 |
| **10** | [GO:0000070](http://amigo.geneontology.org/cgi-bin/amigo/term-details.cgi?term=GO:0000070) | mitotic sister chromatid segregation | 8,00E-09 |
| **11** | [GO:0006260](http://amigo.geneontology.org/cgi-bin/amigo/term-details.cgi?term=GO:0006260) | DNA replication | 8,00E-09 |
| **12** | [GO:0000819](http://amigo.geneontology.org/cgi-bin/amigo/term-details.cgi?term=GO:0000819) | sister chromatid segregation | 1,00E-08 |
| **13** | [GO:0090304](http://amigo.geneontology.org/cgi-bin/amigo/term-details.cgi?term=GO:0090304) | nucleic acid metabolic process | 3,00E-08 |
| **14** | [GO:0010564](http://amigo.geneontology.org/cgi-bin/amigo/term-details.cgi?term=GO:0010564) | regulation of cell cycle process | 9,00E-08 |
| **15** | [GO:0044260](http://amigo.geneontology.org/cgi-bin/amigo/term-details.cgi?term=GO:0044260) | cellular macromolecule metabolic process | 9,00E-08 |
| **16** | [GO:0033554](http://amigo.geneontology.org/cgi-bin/amigo/term-details.cgi?term=GO:0033554) | cellular response to stress | 2,00E-07 |
| **17** | [GO:0051276](http://amigo.geneontology.org/cgi-bin/amigo/term-details.cgi?term=GO:0051276) | chromosome organization | 2,00E-07 |
| **18** | [GO:0006139](http://amigo.geneontology.org/cgi-bin/amigo/term-details.cgi?term=GO:0006139) | nucleobase, nucleoside, nucleotide and nucleic acid metabolic process | 4,00E-07 |
| **19** | [GO:0007098](http://amigo.geneontology.org/cgi-bin/amigo/term-details.cgi?term=GO:0007098) | centrosome cycle | 7,00E-07 |
| **20** | [GO:0000075](http://amigo.geneontology.org/cgi-bin/amigo/term-details.cgi?term=GO:0000075) | cell cycle checkpoint | 8,00E-07 |
| **21** | [GO:0051640](http://amigo.geneontology.org/cgi-bin/amigo/term-details.cgi?term=GO:0051640) | organelle localization | 1,00E-06 |
| **22** | [GO:0040001](http://amigo.geneontology.org/cgi-bin/amigo/term-details.cgi?term=GO:0040001) | establishment of mitotic spindle localization | 1,00E-06 |
| **23** | [GO:0051293](http://amigo.geneontology.org/cgi-bin/amigo/term-details.cgi?term=GO:0051293) | establishment of spindle localization | 2,00E-06 |
| **24** | [GO:0051653](http://amigo.geneontology.org/cgi-bin/amigo/term-details.cgi?term=GO:0051653) | spindle localization | 2,00E-06 |
| **25** | [GO:0051726](http://amigo.geneontology.org/cgi-bin/amigo/term-details.cgi?term=GO:0051726) | regulation of cell cycle | 2,00E-06 |
| **26** | [GO:0016043](http://amigo.geneontology.org/cgi-bin/amigo/term-details.cgi?term=GO:0016043) | cellular component organization | 3,00E-06 |
| **27** | [GO:0034641](http://amigo.geneontology.org/cgi-bin/amigo/term-details.cgi?term=GO:0034641) | cellular nitrogen compound metabolic process | 4,00E-06 |
| **28** | [GO:0006261](http://amigo.geneontology.org/cgi-bin/amigo/term-details.cgi?term=GO:0006261) | DNA-dependent DNA replication | 5,00E-06 |
| **29** | [GO:0007052](http://amigo.geneontology.org/cgi-bin/amigo/term-details.cgi?term=GO:0007052) | mitotic spindle organization | 9,00E-06 |
| **30** | [GO:0006302](http://amigo.geneontology.org/cgi-bin/amigo/term-details.cgi?term=GO:0006302) | double-strand break repair | 1,00E-05 |
| **31** | [GO:0051297](http://amigo.geneontology.org/cgi-bin/amigo/term-details.cgi?term=GO:0051297) | centrosome organization | 1,00E-05 |
| **32** | [GO:0000132](http://amigo.geneontology.org/cgi-bin/amigo/term-details.cgi?term=GO:0000132) | establishment of mitotic spindle orientation | 1,00E-05 |
| **33** | [GO:0051294](http://amigo.geneontology.org/cgi-bin/amigo/term-details.cgi?term=GO:0051294) | establishment of spindle orientation | 1,00E-05 |
| **34** | [GO:0007010](http://amigo.geneontology.org/cgi-bin/amigo/term-details.cgi?term=GO:0007010) | cytoskeleton organization | 1,00E-05 |
| **35** | [GO:0009987](http://amigo.geneontology.org/cgi-bin/amigo/term-details.cgi?term=GO:0009987) | cellular process | 1,00E-05 |
| **36** | [GO:0006310](http://amigo.geneontology.org/cgi-bin/amigo/term-details.cgi?term=GO:0006310) | DNA recombination | 2,00E-05 |
| **37** | [GO:0006807](http://amigo.geneontology.org/cgi-bin/amigo/term-details.cgi?term=GO:0006807) | nitrogen compound metabolic process | 2,00E-05 |
| **38** | [GO:0031023](http://amigo.geneontology.org/cgi-bin/amigo/term-details.cgi?term=GO:0031023) | microtubule organizing center organization | 2,00E-05 |
| **39** | [GO:0046599](http://amigo.geneontology.org/cgi-bin/amigo/term-details.cgi?term=GO:0046599) | regulation of centriole replication | 2,00E-05 |
| **40** | [GO:0050000](http://amigo.geneontology.org/cgi-bin/amigo/term-details.cgi?term=GO:0050000) | chromosome localization | 2,00E-05 |
| **41** | [GO:0051303](http://amigo.geneontology.org/cgi-bin/amigo/term-details.cgi?term=GO:0051303) | establishment of chromosome localization | 2,00E-05 |
| **42** | [GO:0043170](http://amigo.geneontology.org/cgi-bin/amigo/term-details.cgi?term=GO:0043170) | macromolecule metabolic process | 2,00E-05 |
| **43** | [GO:0051656](http://amigo.geneontology.org/cgi-bin/amigo/term-details.cgi?term=GO:0051656) | establishment of organelle localization | 3,00E-05 |
| **44** | [GO:0007080](http://amigo.geneontology.org/cgi-bin/amigo/term-details.cgi?term=GO:0007080) | mitotic metaphase plate congression | 4,00E-05 |
| **45** | [GO:0051321](http://amigo.geneontology.org/cgi-bin/amigo/term-details.cgi?term=GO:0051321) | meiotic cell cycle | 5,00E-05 |
| **46** | [GO:0051983](http://amigo.geneontology.org/cgi-bin/amigo/term-details.cgi?term=GO:0051983) | regulation of chromosome segregation | 7,00E-05 |
| **47** | [GO:0007018](http://amigo.geneontology.org/cgi-bin/amigo/term-details.cgi?term=GO:0007018) | microtubule-based movement | 7,00E-05 |
| **48** | [GO:0006271](http://amigo.geneontology.org/cgi-bin/amigo/term-details.cgi?term=GO:0006271) | DNA strand elongation involved in DNA replication | 1,00E-04 |
| **49** | [GO:0034501](http://amigo.geneontology.org/cgi-bin/amigo/term-details.cgi?term=GO:0034501) | protein localization to kinetochore | 1,00E-04 |
| **50** | [GO:0007346](http://amigo.geneontology.org/cgi-bin/amigo/term-details.cgi?term=GO:0007346) | regulation of mitotic cell cycle | 1,00E-04 |
| **51** | [GO:0000910](http://amigo.geneontology.org/cgi-bin/amigo/term-details.cgi?term=GO:0000910) | Cytokinesis | 1,00E-04 |
| **52** | [GO:0051310](http://amigo.geneontology.org/cgi-bin/amigo/term-details.cgi?term=GO:0051310) | metaphase plate congression | 1,00E-04 |
| **53** | [GO:0006397](http://amigo.geneontology.org/cgi-bin/amigo/term-details.cgi?term=GO:0006397) | mRNA processing | 2,00E-04 |
| **54** | [GO:0007126](http://amigo.geneontology.org/cgi-bin/amigo/term-details.cgi?term=GO:0007126) | Meiosis | 2,00E-04 |
| **55** | [GO:0051327](http://amigo.geneontology.org/cgi-bin/amigo/term-details.cgi?term=GO:0051327) | M phase of meiotic cell cycle | 2,00E-04 |
| **56** | [GO:0044237](http://amigo.geneontology.org/cgi-bin/amigo/term-details.cgi?term=GO:0044237) | cellular metabolic process | 3,00E-04 |
| **57** | [GO:0010824](http://amigo.geneontology.org/cgi-bin/amigo/term-details.cgi?term=GO:0010824) | regulation of centrosome duplication | 3,00E-04 |
| **58** | [GO:0006270](http://amigo.geneontology.org/cgi-bin/amigo/term-details.cgi?term=GO:0006270) | DNA-dependent DNA replication initiation | 3,00E-04 |
| **59** | [GO:0010212](http://amigo.geneontology.org/cgi-bin/amigo/term-details.cgi?term=GO:0010212) | response to ionizing radiation | 4,00E-04 |
| **60** | [GO:0051298](http://amigo.geneontology.org/cgi-bin/amigo/term-details.cgi?term=GO:0051298) | centrosome duplication | 4,00E-04 |
| **61** | [GO:0033044](http://amigo.geneontology.org/cgi-bin/amigo/term-details.cgi?term=GO:0033044) | regulation of chromosome organization | 4,00E-04 |
| **62** | [GO:0032886](http://amigo.geneontology.org/cgi-bin/amigo/term-details.cgi?term=GO:0032886) | regulation of microtubule-based process | 5,00E-04 |
| **63** | [GO:0046605](http://amigo.geneontology.org/cgi-bin/amigo/term-details.cgi?term=GO:0046605) | regulation of centrosome cycle | 5,00E-04 |
| **64** | [GO:0000236](http://amigo.geneontology.org/cgi-bin/amigo/term-details.cgi?term=GO:0000236) | mitotic prometaphase | 6,00E-04 |
| **65** | [GO:0051299](http://amigo.geneontology.org/cgi-bin/amigo/term-details.cgi?term=GO:0051299) | centrosome separation | 6,00E-04 |
| **66** | [GO:0006297](http://amigo.geneontology.org/cgi-bin/amigo/term-details.cgi?term=GO:0006297) | nucleotide-excision repair, DNA gap filling | 7,00E-04 |
| **67** | [GO:0007062](http://amigo.geneontology.org/cgi-bin/amigo/term-details.cgi?term=GO:0007062) | sister chromatid cohesion | 7,00E-04 |
| **68** | [GO:0007099](http://amigo.geneontology.org/cgi-bin/amigo/term-details.cgi?term=GO:0007099) | centriole replication | 8,00E-04 |
| **69** | [GO:0022616](http://amigo.geneontology.org/cgi-bin/amigo/term-details.cgi?term=GO:0022616) | DNA strand elongation | 8,00E-04 |
| **70** | [GO:0031124](http://amigo.geneontology.org/cgi-bin/amigo/term-details.cgi?term=GO:0031124) | mRNA 3'-end processing | 9,00E-04 |
| **71** | [GO:0033043](http://amigo.geneontology.org/cgi-bin/amigo/term-details.cgi?term=GO:0033043) | regulation of organelle organization | 1,00E-03 |
| **72** | [GO:0006259](http://amigo.geneontology.org/cgi-bin/amigo/term-details.cgi?term=GO:0006259) | DNA metabolic process | <2e-16 |
| **73** | [GO:0048285](http://amigo.geneontology.org/cgi-bin/amigo/term-details.cgi?term=GO:0048285) | organelle fission | <2e-16 |
| **74** | [GO:0022403](http://amigo.geneontology.org/cgi-bin/amigo/term-details.cgi?term=GO:0022403) | cell cycle phase | <2e-16 |
| **75** | [GO:0000280](http://amigo.geneontology.org/cgi-bin/amigo/term-details.cgi?term=GO:0000280) | nuclear division | <2e-16 |
| **76** | [GO:0007067](http://amigo.geneontology.org/cgi-bin/amigo/term-details.cgi?term=GO:0007067) | Mitosis | <2e-16 |
| **77** | [GO:0000087](http://amigo.geneontology.org/cgi-bin/amigo/term-details.cgi?term=GO:0000087) | M phase of mitotic cell cycle | <2e-16 |
| **78** | [GO:0007049](http://amigo.geneontology.org/cgi-bin/amigo/term-details.cgi?term=GO:0007049) | cell cycle | <2e-16 |
| **79** | [GO:0000279](http://amigo.geneontology.org/cgi-bin/amigo/term-details.cgi?term=GO:0000279) | M phase | <2e-16 |
| **80** | [GO:0022402](http://amigo.geneontology.org/cgi-bin/amigo/term-details.cgi?term=GO:0022402) | cell cycle process | <2e-16 |
| **81** | [GO:0007127](http://amigo.geneontology.org/cgi-bin/amigo/term-details.cgi?term=GO:0007127) | meiosis I | 0.001 |
| **82** | [GO:0006412](http://amigo.geneontology.org/cgi-bin/amigo/term-details.cgi?term=GO:0006412) | Translation | 0.001 |
| **83** | [GO:0000724](http://amigo.geneontology.org/cgi-bin/amigo/term-details.cgi?term=GO:0000724) | double-strand break repair via homologous recombination | 0.001 |
| **84** | [GO:0000725](http://amigo.geneontology.org/cgi-bin/amigo/term-details.cgi?term=GO:0000725) | recombinational repair | 0.001 |
| **85** | [GO:0030010](http://amigo.geneontology.org/cgi-bin/amigo/term-details.cgi?term=GO:0030010) | establishment of cell polarity | 0.001 |
| **86** | [GO:0051716](http://amigo.geneontology.org/cgi-bin/amigo/term-details.cgi?term=GO:0051716) | cellular response to stimulus | 0.001 |
| **87** | [GO:0007143](http://amigo.geneontology.org/cgi-bin/amigo/term-details.cgi?term=GO:0007143) | female meiosis | 0.001 |
| **88** | [GO:0007093](http://amigo.geneontology.org/cgi-bin/amigo/term-details.cgi?term=GO:0007093) | mitotic cell cycle checkpoint | 0.001 |
| **89** | [GO:0000375](http://amigo.geneontology.org/cgi-bin/amigo/term-details.cgi?term=GO:0000375) | RNA splicing, via transesterification reactions | 0.002 |
| **90** | [GO:0006289](http://amigo.geneontology.org/cgi-bin/amigo/term-details.cgi?term=GO:0006289) | nucleotide-excision repair | 0.002 |
| **91** | [GO:0007088](http://amigo.geneontology.org/cgi-bin/amigo/term-details.cgi?term=GO:0007088) | regulation of mitosis | 0.002 |
| **92** | [GO:0051783](http://amigo.geneontology.org/cgi-bin/amigo/term-details.cgi?term=GO:0051783) | regulation of nuclear division | 0.002 |
| **93** | [GO:0070507](http://amigo.geneontology.org/cgi-bin/amigo/term-details.cgi?term=GO:0070507) | regulation of microtubule cytoskeleton organization | 0.002 |
| **94** | [GO:0000077](http://amigo.geneontology.org/cgi-bin/amigo/term-details.cgi?term=GO:0000077) | DNA damage checkpoint | 0.002 |
| **95** | [GO:0016071](http://amigo.geneontology.org/cgi-bin/amigo/term-details.cgi?term=GO:0016071) | mRNA metabolic process | 0.002 |
| **96** | [GO:0045132](http://amigo.geneontology.org/cgi-bin/amigo/term-details.cgi?term=GO:0045132) | meiotic chromosome segregation | 0.002 |
| **97** | [GO:0045292](http://amigo.geneontology.org/cgi-bin/amigo/term-details.cgi?term=GO:0045292) | nuclear mRNA cis splicing, via spliceosome | 0.002 |
| **98** | [GO:0006396](http://amigo.geneontology.org/cgi-bin/amigo/term-details.cgi?term=GO:0006396) | RNA processing | 0.002 |
| **99** | [GO:0034621](http://amigo.geneontology.org/cgi-bin/amigo/term-details.cgi?term=GO:0034621) | cellular macromolecular complex subunit organization | 0.003 |
| **100** | [GO:0044238](http://amigo.geneontology.org/cgi-bin/amigo/term-details.cgi?term=GO:0044238) | primary metabolic process | 0.003 |
